# Biogeography and evolutionary history of *Puntius* sensu lato (Teleostei: Cyprinidae) in Sri Lanka

**DOI:** 10.1038/s41598-023-45377-9

**Published:** 2023-10-31

**Authors:** Hiranya Sudasinghe, Tharindu Ranasinghe, Neelesh Dahanukar, Rajeev Raghavan, Lukas Rüber, Rohan Pethiyagoda, Madhava Meegaskumbura

**Affiliations:** 1 Evolutionary Ecology and Systematics Laboratory, Department of Molecular Biology and Biotechnology, University of Peradeniya, Peradeniya, 20400 Sri Lanka; 2 Postgraduate Institute of Science, University of Peradeniya, Peradeniya, 20400 Sri Lanka; 3 Evolutionary Ecology, Institute of Ecology and Evolution, University of Bern, 3012 Bern, Switzerland; 4 Naturhistorisches Museum Bern, Bernastrasse, 15, 3005 Bern, Switzerland; 5 Wild Island Foundation, 6A, Mendis Lane, Moratuwa, 10400 Sri Lanka; 6 Department of Life Sciences, School of Natural Sciences, Shiv Nadar Institution of Eminence, Delhi, India; 7 Department of Fisheries Resource Management, Kerala University of Fisheries and Ocean Studies (KUFOS), Kochi, India; 8 Aquatic Ecology and Evolution, Institute of Ecology and Evolution, University of Bern, 3012 Bern, Switzerland; 9 Ichthyology Section, Australian Museum, 1 William Street, Sydney, NSW 2010 Australia; 10 Guangxi Key Laboratory for Forest Ecology and Conservation, College of Forestry, Guangxi University, Nanning, 530004 Guangxi People’s Republic of China

**Keywords:** Evolution, Evolutionary genetics, Phylogenetics, Population genetics, Speciation, Taxonomy

## Abstract

Sri Lanka’s biota is derived largely from Southeast Asian lineages which immigrated via India following its early-Eocene contact with Laurasia. The island is now separated from southeastern India by the 30 km wide Palk Strait which, during sea-level low-stands, was bridged by the 140 km-wide Palk Isthmus. Consequently, biotic ingress and egress were mediated largely by the climate of the isthmus. Because of their dependence on perennial aquatic habitats, freshwater fish are useful models for biogeographic studies. Here we investigate the timing and dynamics of the colonization of—and diversification on—Sri Lanka by a group of four closely-related genera of cyprinid fishes (*Puntius* sensu lato). We construct a molecular phylogeny based on two mitochondrial and two nuclear gene markers, conduct divergence timing analyses and ancestral-range estimations to infer historical biogeography, and use haplotype networks to discern phylogeographic patterns. The origin of *Puntius* s.l. is dated to ~ 20 Ma. The source of diversification of *Puntius* s.l. is Sri Lanka-Peninsular India. Species confined to perhumid rainforests show strong phylogeographic structure, while habitat generalists show little or no such structure. Ancestral range estimations for *Plesiopuntius bimaculatus* and *Puntius dorsalis* support an ‘Out of Sri Lanka’ scenario. Sri Lankan *Puntius* s.l. derive from multiple migrations across the Palk Isthmus between the early Miocene and the late Pleistocene. Species dependent on an aseasonal climate survived aridification in rainforest refugia in the island’s perhumid southwest and went on to recolonize the island and even southern India when pluvial conditions resumed. Our results support an historical extinction of Sri Lanka’s montane aquatic fauna, followed by a recent partial recolonization of the highlands, showing also that headwater stream capture facilitated dispersal across basin boundaries.

## Introduction

The tropical continental island of Sri Lanka is separated from Southeast India by the ~ 30 km-wide Palk Strait, a shallow-shelf sea. The strait was bridged by an Isthmus during periods of lowered sea level between the late Oligocene and early Holocene^1^. This land bridge, known as the Palk Isthmus^2^, was the only terrestrial route by which plants and animals could disperse between Sri Lanka and India. Bossuyt et al.^3^ showed that despite the Isthmus being emergent for almost the entirety of the Plio-Pleistocene, instances of biotic exchange between Sri Lanka and India have been rare. Pethiyagoda and Sudasinghe^1^ argued that except during short intervals, the climate of the Isthmus was too arid and too strongly seasonal to facilitate biotic dispersal between the rainforests of the Western Ghats mountains of southwest India and those of southwest Sri Lanka.

Despite prolonged terrestrial connectivity with India, clade-level endemism among Sri Lankan vertebrates is uncommon^3^. Further, despite the island’s Gondwanan origins, the Gondwanan signature in its fauna and flora is faint, suggestive of a major extinction some time prior to the Oligocene: only a handful of putatively Gondwanan relict taxa occurs on the island^1^. Thus it is that Sri Lanka’s biodiversity is derived largely from lineages that entered India subsequent to its contacts with—and eventual accretion to—Asia starting ~ 52 Ma^4^, dispersed down the peninsula, and went on to immigrate via the Palk Isthmus. In the case of organisms associated with a perhumid climate, however, opportunities to enter Sri Lanka appear to have been rare. Most of the island’s vertebrate endemics may be attributed to autochthonous diversifications that stem from ancestors associated with aseasonal rainfall patterns, which immigrated via the Palk Isthmus during pluvial phases during the late Oligocene to early Miocene^1^. The more recent colonizations involve dry-adapted taxa which dispersed across the Palk Isthmus during the Plio-Pleistocene. Meegaskumbura et al.^5^ show, for example, that the diversification on Sri Lanka of some 48 species of the shrub-frog genus *Pseudophilautus*, almost all of which inhabit the island’s perhumid southwestern quarter, is monophyletic, deriving from a common ancestor dating to ca. 25 (95% HPD = 21.9–45.1) Ma. Beenaerts et al.^6^, meanwhile, estimated the diversification of 51 endemic species of gecarcinucid freshwater crabs to date to an ancestor that colonized Sri Lanka around 7.4 (95% HPD = 4.6–11.4) Ma.

Because they can disperse only via rivers and streams, and not, for example, by overseas rafting, freshwater organisms are useful targets for biogeographic studies. Among the ~ 100 fish species that obligatorily spend part of their life cycle in freshwaters in Sri Lanka, about half are habitat generalists shared between the island’s lowland floodplains and southeast India, while the rest are endemic, largely confined to the rainforests of the island’s perhumid south-western wet zone (annual rainfall > 2 m)^1^. This habitat association, together with the available timing studies, suggest that the generalist freshwater fish fauna derives from multiple Plio-Pleistocene colonization events across the Palk Isthmus^7–10^.

The autochthonous diversifications of freshwater fishes on Sri Lanka, however, show relatively greater antiquity. The endemic diversifications within the cypriniform genera *Systomus*, *Rasbora*, and *Devario*, for instance, have mean crown ages of 22.4 (95% HPD = 16.6–27.9) Ma, 10.0 (7.1–13.3) Ma, and 4.2 (2.9–5.4) Ma, respectively^8–10^*.*

The genus *Puntius* is part of the cyprinid subfamily Smiliogastrinae, which contains about 470 valid species of small to medium-sized freshwater fishes distributed through much of tropical and subtropical Asia and Africa. The Asian smiliogastrine genera show a marked division east and west of the Indo-Burman Ranges (hereafter IBR). Although the species diversity of *Puntius* is richest in the Indian subcontinent, a few members occur to the east of IBR. Sudasinghe et al.^11^ investigated the systematics of this group and showed that the seven Sri Lankan species until then assigned to *Puntius* in fact belong to four morphologically and phylogenetically distinct lineages, to reflect which they established three new monotypic genera: *Rohanella* (represented by *R. titteya* (Deraniyagala)), a Sri Lankan endemic; *Bhava* (represented by* B. vittata* (Day)), and *Plesiopuntius* (represented by *Pl. bimaculatus* Bleeker). *Bhava* and *Plesiopuntius* occur in Sri Lanka as well as southern India. *Puntius*, meanwhile, now comprises some 28 species^11^.

The Sri Lankan members of *Puntius* sensu lato (s.l.) comprise generalists that occur in a variety of habitats in both the dry and wet zones of the island, as well as habitat specialists confined mostly to the perhumid zone. Three are endemics (*Puntius kamalika* Silva, Maduwage, & Pethiyagoda*, P. kelumi* Pethiyagoda, Silva, Maduwage, & Meegaskumbura*,* and *Rohanella titteya* (Deraniyagala)), of which the latter two are confined to the southwestern wet zone, while the former is distributed in both the wet zone and the northern dry zone lowlands^1,11^. The remaining four species are shared between Sri Lanka and southern India (*Bhava vittata* (Day), *P. dorsalis* (Jerdon), *P. thermalis* (Valenciennes), and *Plesiopuntius bimaculatus* (Bleeker)). Sudasinghe et al.^11^ further identified distinct evolutionary lineages within *P. dorsalis*, *P. kelumi* and *Plesiopuntius bimaculatus,* revealing previously unrecognized genetic diversity. Here, building on their molecular phylogeny based on analyses of the mitochondrial cytochrome b (*cytb*) and cytochrome oxidase subunit 1 (*cox1*), the nuclear recombination activating protein 1 (*rag1*), and interphotoreceptor retinoid-binding protein (*irbp*) gene markers, and by the inclusion of newly generated sequences from India, we attempt to elucidate the biogeography and phylogeography of *Puntius* s.l. in Sri Lanka.

In particular, we seek to test several hypotheses: (a) As in the case of other highly derived vertebrate groups associated with the wet zone (such as the *Pseudophilautus* shrub frogs and the endemic clade of *Systomus*^5,9^), *Rohanella* diverged from *Plesiopuntius*, its sister group, in the late Oligocene or early Miocene; (b) as posited for the flora by Ashton^12^, Sri Lanka’s freshwater fishes too, retreated into evergreen rainforest refugia in perhumid valleys as the monsoons weakened during Pleistocene glacial maxima, going on to colonize the island once more when the climate became less strongly seasonal; (c) as proposed by Bose et al.^13^, some lineages that benefited from these evergreen rainforest refugia back-migrated to India when pluvial conditions resumed on the Palk Isthmus; (d) as hypothesized by Pethiyagoda and Sudasinghe^1^, the fishes of the Sri Lankan highlands suffered a general extinction, predicting that the population of the only member of *Puntius* s.l. present in the highlands should have colonized that region recently; (e) notwithstanding Sri Lanka’s tectonic stability, headwater stream capture has operated to facilitate the dispersal of fishes across watersheds; and (f) as hypothesized by Pethiyagoda and Sudasinghe^1^, fishes occurring in Sri Lanka’s northern dry zone are shared with the southern plains of the Indian peninsula.

## Results

### Molecular phylogeny

The phylogenetic relationships recovered from the ML analysis for the combined dataset were congruent with those of Sudasinghe et al.^11^. The three species groups, P. sophore, P. mahecola, and P. dorsalis within *Puntius* s.s. were recovered as monophyletic with high node support (Fig. S1, BP = 100). The P. sophore group was recovered as the sister group of the [P. dorsalis + P. mahecola] group, with high node support (BP = 87). Within *Puntius* s.l., *Plesiopuntius* (the Pl. bimaculatus species group) and *Rohanella* (the R. titteya species group) show a well-supported sister-group relationship (BP = 100); it is recovered as the sister group of *Puntius* s.s. (BP = 92). *Bhava* (the B. vittata species group) is recovered as the sister group of [*Puntius* s.s. + [Pl. bimaculatus group + *R. titteya*]] (BP = 85).

Eight subclades are recovered within the P. sophore group, which is the most widespread of the species groups within *Puntius* s.s. The newly generated sequences from India for the P. sophore group confirm the taxonomic identity of subclades 2 and 4 as *Puntius sophore* (Hamilton) and *P. terio* (Hamilton), respectively, while also confirming that *P. thermalis* (subclade 6) is shared between Sri Lanka and southern India as hypothesized^11^. Within the P. mahecola group, *P. kamalika* and *P. mahecola* (Valenciennes) show a well-supported sister-group relationship (BP = 100). The P. dorsalis group is represented by *P. dorsalis* and *P. kelumi,* which show a well-supported sister-group relationship (BP = 100). Within both *P. dorsalis* and *P. kelumi*, three subclades are recovered, congruent with Sudasinghe et al.^11^. Three subclades are recovered also within the Pl. bimaculatus group, the inclusion of the newly generated sequences from India confirm that subclade 3 is shared between Sri Lanka and mainland, as hypothesized^11^.

### Divergence timing and ancestral range estimation

**Figure 1:**
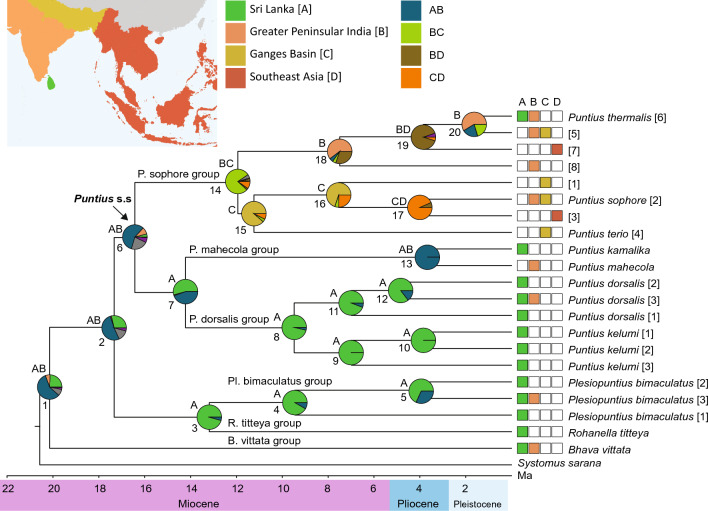
Bayesian time-calibrated tree, based on the *cytb* substitution rate, for the concatenated mitochondrial + nuclear (3964 bp, 22 taxa) dataset of *Puntius* s.l. Pies at each node represent the ancestral-range estimations of *Puntius* s.l., using the DEC model. Most likely ancestral ranges immediately before a speciation event are indicated above each node. Numbers below nodes refer to the node identifiers in Table 1.

The divergence-timing analysis for the 22-taxa dataset using a *cytb* substitution rate in BEAST estimated the divergence of *Bhava* from the remaining [*Puntius* s.l. + *Puntius* s.s.] at 20.1 Ma (95% HPD: 17.0–23.4 Ma), in the early Miocene (Fig. 1, Table 1). The divergence of [*Plesiopuntius* + *Rohanella*] from *Puntius* s.s. was estimated at 17.3 Ma (95% HPD: 14.8–20.0 Ma) in the early Miocene. The crown age of the diversification of *Puntius* s.s. was estimated at 16.1 Ma (95% HPD: 13.8–18.7 Ma), in the mid-Miocene. The divergence between the P. mahecola and P. dorsalis groups was estimated at 14.2 Ma (95% HPD: 11.8–16.7 Ma), also in the mid-Miocene. The crown ages for the diversifications of the P. sophore and P. dorsalis groups were estimated at 11.9 Ma (95% HPD: 9.8–14.1 Ma) and 9.4 Ma (95% HPD: 7.7–11.2), respectively, in the mid- to late Miocene. The divergence between *Rohanella* and *Plesiopuntius* was estimated at 13.1 Ma (95% HPD: 10.7–15.7 Ma), in the mid-Miocene. The divergence between *P. kamalika* and *P. mahecola*, within the P. mahecola group, was estimated at 3.6 Ma (95% HPD: 2.4–5.0 Ma) in the Plio-Pleistocene.

**Table 1:** Comparison of the mean, 95% highest posterior density (HPD), posterior probability (PP) of the divergence timing analysis, and distributions suggested by the ancestral-range reconstruction analysis.

Node	PP	Mean (Ma)	95% HPD (Ma)	Ancestral range
1	1.0	20.1	17.0–23.4	AB
2	1.0	17.3	14.8–20.0	AB
3	1.0	13.1	10.7–15.7	A
4	1.0	9.4	7.3–11.6	A
5	1.0	3.9	2.7–5.2	A
6	0.7	16.1	13.8–18.7	AB
7	1.0	14.2	11.8–16.7	A
8	1.0	9.4	7.7–11.2	A
9	1.0	6.9	5.1–8.8	A
10	1.0	3.7	2.4–4.9	A
11	1.0	6.9	5.4–8.6	A
12	1.0	4.8	3.4–6.2	A
13	1.0	3.6	2.4–5.0	AB
14	1.0	11.9	9.8–14.1	BC
15	0.4	10.2	8.2–12.4	C
16	1.0	7.5	5.2–9.9	C
17	1.0	3.9	2.2–5.8	CD
18	1.0	7.4	5.4–9.6	B
19	1.0	3.8	2.8–4.9	BD
20	1.0	1.6	1.0–2.2	B

The reconstruction of ancestral ranges of *Puntius* s.l. was evaluated using the DEC and DEC + J models, under AICc. DEC + J did not improve the model. Hence, we report only the results from the DEC model. The ancestral ranges in each scenario, together with the vicariance and dispersal events, are shown in Fig. 1 and Table 1. The common ancestor of *Puntius* s.l. was inferred to be a widespread species that occurred in both Sri Lanka and India (Fig. 1). The common ancestors of the P. sophore, P. mahecola, and P. dorsalis groups were estimated to have been distributed in Peninsular India and the Ganges Basin; Sri Lanka and India; and Sri Lanka, respectively (Fig. 1). The most probable distribution of the common ancestor of the [P. mahecola + P. dorsalis] species groups and [*Rohanella* + *Plesiopuntius*] was estimated to be Sri Lanka. The common ancestor *of P. kamalika* and *P. mahecola* was estimated to be a species widespread in Sri Lanka and India, suggesting a vicariance event separating the two species during the Plio-Pleistocene. Within the P. dorsalis group and *Plesiopuntius*, a Sri Lanka-to-India dispersal event is evident in the common ancestor of subclade 3, during the Plio-Pleistocene. By contrast, in the case of *P. thermalis*, an out of India (to Sri Lanka) dispersal event appears to have occurred during the Plio-Pleistocene. Within the P. sophore group, it appears that there were two independent dispersal events out of India (India + Ganges Basin) to Southeast Asia, both during the late Miocene.

### Genetic diversity and phylogeography

For each gene marker (*cytb*, *cox1*, and *rag1)*, the number of haplotypes, polymorphic sites, parsimony-informative sites, and nucleotide and haplotype diversities, are given in Supplementary Table S1. None of the neutrality tests were significant for any of the Sri Lankan species of *Puntius* s.l. Overall, for all the gene markers, the nucleotide diversities were greater in *P. dorsalis*, *P. kelumi*, and *Plesiopuntius bimaculatus*, compared to *P. thermalis*, *P. kamalika, Rohanella titteya*, and *B. vittata,* while the haplotype diversities were similar for all species (Supplementary Table S1). The former three species also show stronger phylogeographic signals than the rest.

**Figure 2:**
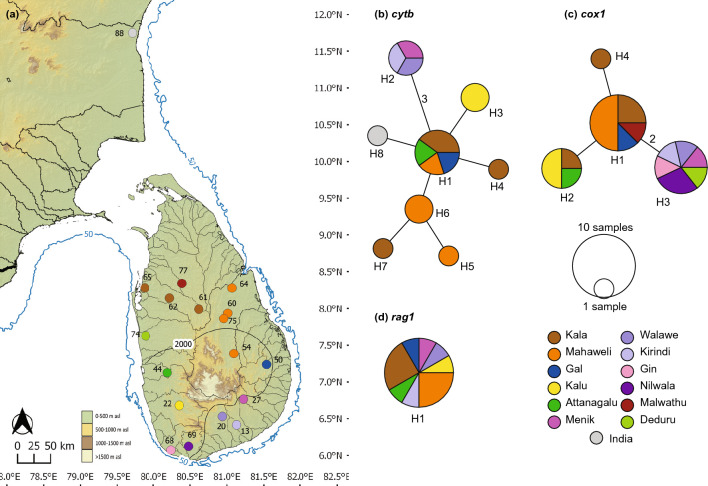
(**a**) Indication of the geographical origin of samples of *Puntius thermalis* used in the molecular analysis. Numbers on the map represent the sampling localities listed in Supplementary Table S3. Median-joining haplotype networks for *P. thermalis* based on the analysis of (**b**) a 1073 bp fragment of the *cytb* gene, (**c**) a 630 bp fragment of the *cox1* gene, and (**d**) a 1470 bp fragment of the *rag1* gene. The number of mutational steps > 1 is shown. Legend colors correspond to river basins. The bold black line in Sri Lanka indicates the 2000-mm isohyet, which encompasses the wet zone. The 50 m isobath (dark blue) is based on GEBCO Compilation Group (2020). Major river basins in Sri Lanka are labelled in black.

*Puntius thermalis* (Fig. 2) contains eight *cytb* haplotypes: H1-H7 confined to Sri Lanka, and H8 from southern India (Fig. 2b). H1 is widespread, present in the Attanagalu, Kala, Gal, and Mahaweli basins, which traverse the lowlands of both the wet and the dry zones. Meanwhile, H2 is confined to the contiguous east-flowing basins Walawe, Kirindi, and Kumbukkan. The remaining five Sri Lankan *cytb* haplotypes are each confined to individual basins (H5, H6 to the Mahaweli; H4, H7 to the Kala; and H3 to the Kalu), but without a strong phylogeographic structure. The *cox1* network of *P. thermalis* consists of four haplotypes (Fig. 2c). Except for H4, which is confined to the Kala basin, the rest are widespread in the lowland floodplains of both the wet and the dry zones; these too, do not exhibit a clear phylogeographic structure. The *rag1* network of *P. thermalis* consists of only a single ubiquitous haplotype (Fig. 2d).

**Figure 3:**
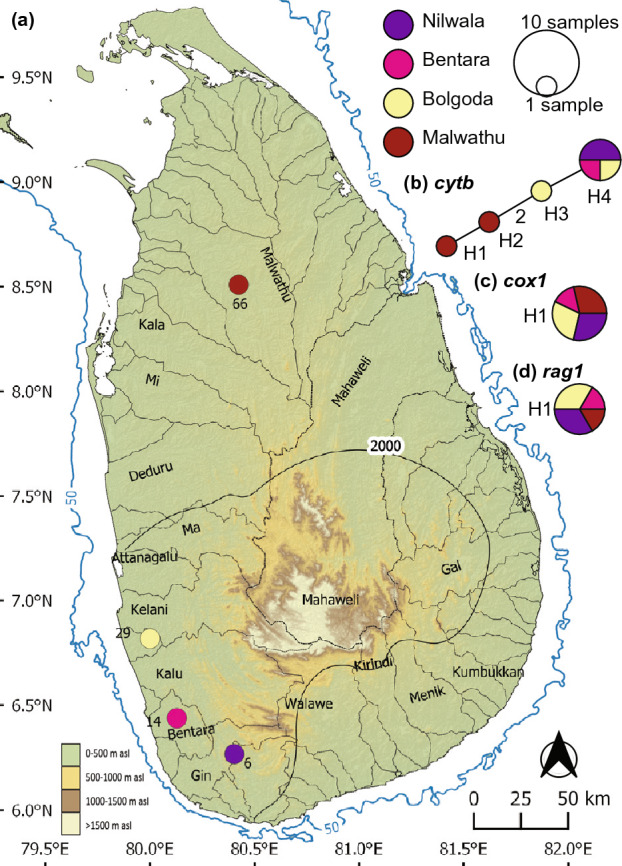
(**a**) Indication of the geographical origin of samples of *Puntius kamalika* used in the molecular analysis. Numbers on the map represent the sampling localities listed in Supplementary Table S3. Median-joining haplotype networks for *P. kamalika* based on the analysis of (**b**) a 1073 bp fragment of the *cytb* gene, (**c**) a 630 bp fragment of the *cox1* gene, and (**d**) a 1470 bp fragment of the *rag1* gene. The number of mutational steps > 1 is shown. Legend colors correspond to river basins. The bold black line in Sri Lanka indicates the 2000-mm isohyet, which encompasses the wet zone. The 50 m isobath (dark blue) is based on GEBCO Compilation Group (2020). Major river basins in Sri Lanka are labelled in black.

*Puntius kamalika* (Fig. 3) contains only four *cytb* haplotypes (Fig. 3b), among which H4 is shared between the perhumid Bolgoda, Bentara, and Nilwala basins. The haplotypes H1 and H2 are confined to the Malwathu basin in the northern dry zone, while H3 and H4 are confined to the southwestern wet zone (Fig. 3b). The dry and wet zone haplotypes are separated by a minimum of just 2 mutational steps: no strong phylogeographic structure is evident. The *cox1* and *rag1* networks of *P. kamalika* exhibit only a single haplotype, shared between the dry zone and wet zone populations (Fig. 3c,d).

**Figure 4:**
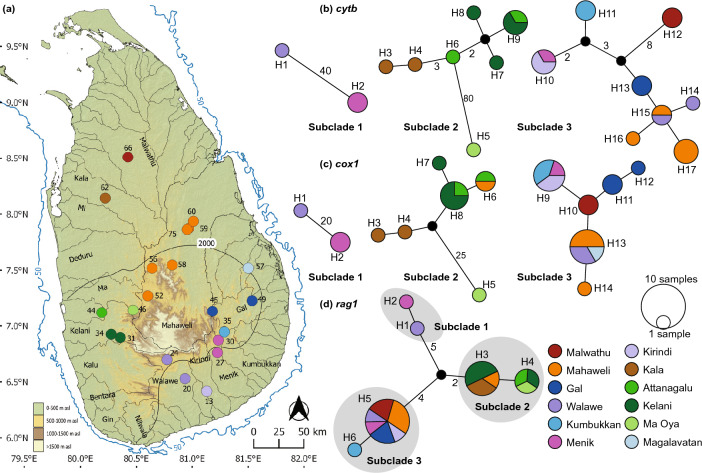
(**a**) Indication of the geographical origin of samples of *Puntius dorsalis* used in the molecular analysis. Numbers on the map represent the sampling localities listed in Supplementary Table S3. Median-joining haplotype networks for *P. dorsalis* based on the analysis of (**b**) a 1073 bp fragment of the *cytb* gene, (**c**) a 630 bp fragment of the *cox1* gene, and (**d**) a 1470 bp fragment of the *rag1* gene. The number of mutational steps > 1 is shown. The black circles are hypothetical nodes. Legend colors correspond to river basins. The bold black line in Sri Lanka indicates the 2000-mm isohyet, which encompasses the wet zone. The 50 m isobath (dark blue) is based on GEBCO Compilation Group (2020). Major river basins in Sri Lanka are labelled in black.

*Puntius dorsalis* (Fig. 4) possesses a total of 17 *cytb* haplotypes (Fig. 4b). Subclade 1 includes two of these (H1-H2), from the headwaters of the southern and southeastern basins (Belihul Oya: Walawe basin, and Badalkumbura: Menik basin). Subclade 2 includes seven haplotypes (H3-H9). Haplotype H5 from Aranayake, in the Ma Oya basin, is separated by a minimum of 80 mutational steps. The remaining haplotypes of subclade 2, from the perhumid zone, are from two contiguous basins: the Attanagalu (H6) and the Kelani (H7-H8), with H9 shared between the two basins (H9). The dry zone representatives of subclade 2 from the Kala basin contain two haplotypes, H3 and H4, which are separated from the wet zone haplotypes by a minimum of three mutational steps. All samples of subclade 2 derive from riverine habitats. Subclade 3 is the most widespread within Sri Lanka. It includes eight haplotypes (H10-H17), all originating from both lotic and lentic habitats. Haplotype H12 is from the Malwathu basin in the northern dry zone, while the remainder are from the Mahaweli (H16,H17), and from the southern (Walawe) and eastern (Kirindi, Menik, Kumbukkan, Gal, and Magalavatan) basins. In subclade 3, H10 is shared between two contiguous basins, the Kirindi and Menik, while H15 is shared between the Mahaweli and the Walawe. The *cox1* network of *P. dorsalis* consists of a total of 14 haplotypes (Fig. 4c). The *cox1* phylogeographic structure is similar to that observed for *cytb*. In subclade 2, H6 is shared between the Attanagalu and Knuckles Hills of the Mahaweli basin, a disjunct distribution. The *rag1* network of *P. dorsalis* consists of a total of six haplotypes, with each subclade containing two (Fig. 4d). The phylogeographic structure observed in mitochondrial haplotypes is reflected in the *rag1* haplotype network of *P. dorsalis*, though with the subclades separated by fewer mutational steps.

**Figure 5:**
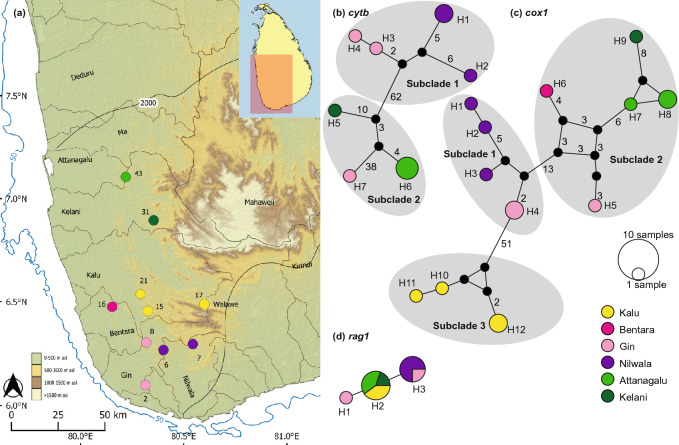
(**a**) Indication of the geographical origin of samples of *Puntius kelumi* used in the molecular analysis. Numbers on the map represent the sampling localities listed in Supplementary Table S3. Median-joining haplotype networks for *P. kelumi* based on the analysis of (**b**) a 1073 bp fragment of the *cytb* gene, (**c**) a 630 bp fragment of the *cox1* gene, and (**d**) a 1470 bp fragment of the *rag1* gene. The number of mutational steps > 1 is shown. The black circles are hypothetical nodes. Legend colors correspond to river basins. The bold black line in Sri Lanka indicates the 2000-mm isohyet, which encompasses the wet zone. The 50 m isobath (dark blue) is based on GEBCO Compilation Group (2020). Major river basins in Sri Lanka are labelled in black.

*Puntius kelumi* (Fig. 5) contains seven *cytb* haplotypes (Fig. 5b). Subclade 1 includes four, two each from Gin (H3-H4) and Nilwala (H1-H2), while subclade 2 includes a haplotype each confined to the Attanagalu (H6), Kelani (H5), and Gin (H7). The *cox1* network of *P. kelumi* (Fig. 5c) consists of a total of 12 haplotypes; its phylogeographic structure is similar to that observed for *cytb*, though with samples from the Bentara basin in subclade 2 (H6) and those from the Kalu forming subclade 3 (H10-H12). The *rag1* network of *P. kelumi* consists of three haplotypes (Fig. 5d), with H3 representing subclade 1 and H1 and H2 representing subclade 2. The phylogeographic structure observed for mitochondrial haplotypes is not apparent for *rag1* in *P. kelumi*, and the three *rag1* haplotypes are separated by only one or two mutational steps.

**Figure 6:**
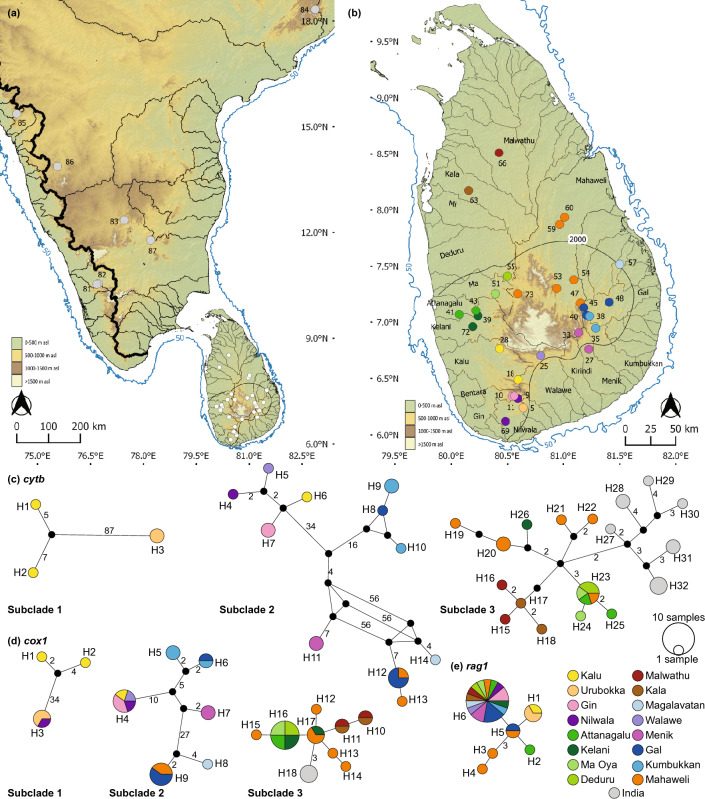
(**a**,**b**) Indication of the geographical origin of samples of *Plesiopuntius bimaculatus* used in the molecular analysis. Numbers on the map represent the sampling localities listed in Supplementary Table S3. Median-joining haplotype networks for *Pl. bimaculatus* based on the analysis of (**c**) a 1073 bp fragment of the *cytb* gene, (**d**) a 630 bp fragment of the *cox1* gene, and (**e**) a 1470 bp fragment of the *rag1* gene. The number of mutational steps > 1 is shown. The black circles are hypothetical nodes. Legend colors correspond to river basins. The bold black line in Sri Lanka indicates the 2000-mm isohyet, which encompasses the wet zone. The bold black line along the axis of the Western Ghats indicates the boundary between east- and west-flowing drainages. The 50 m isobath (dark blue) is based on GEBCO Compilation Group (2020). Major river basins in Sri Lanka are labelled in black.

*Plesiopuntius bimaculatus* (Fig. 6) contains a network of 32 *cytb* haplotypes (Fig. 6b). Subclade 1 contains three haplotypes (H1-H3), from the Kalu and Urubokka basins in the wet zone, a disjunct distribution, while Subclade 2 contains 11 (H4-H14), confined to a group of contiguous river basins. Haplotypes H4-H7 are from the south and southwest basins Nilwala, Kalu, and Gin, respectively forming Subclade 2.iii. Haplotypes H8 (Gal basin), H9-H10 (Kumbukkan), and H11 (Menik), form subclade 2.ii; these eastern basins too, are contiguous. Haplotypes H14 (Magalavatan), H13 (Mahaweli), and H12 (shared between headwaters of the Gal and Mahaweli basins) form subclade 2.i. Subclade 3, which is the most widespread, contains 18 haplotypes (H15-H32). All the samples from India (H27-H32) cluster within subclade 3, which is separated from the remaining Sri Lankan haplotypes by a minimum of four mutational steps (Fig. 3b). Sub-structuring within the Sri Lankan haplotypes is apparent in subclade 3, with H15-H18 confined to the contiguous northern basins Kala and Malwathu; H19-H22, H24, H25, H26 are confined to Mahaweli, Ma, Attanagalu, and Kelani, respectively; and H23 is shared between the contiguous western basins Deduru, Ma, Attanagalu, and a large eastern basin, the Mahaweli. The *cox1* network of *Plesiopuntius bimaculatus* consists of a total of 18 haplotypes, with a phylogeographic structure similar to observed for *cytb* (Fig. 6c). In subclade 1, H3 is shared between the Nilwala and Urubokka basins. In subclade 2, H4 is shared between the contiguous Kalu, Gin, Nilwala, and Walawe basins; H6 is shared between the contiguous Gal and Magalavatan basins; and H9 is shared between the headwaters of the Gal and the Mahaweli. In subclade 3, H10-H11 are shared between the contiguous Kala and Malwathu basins in the northern dry zone; H16 is shared between the contiguous western basins Deduru, Ma, Attanagalu and Kelani; while H17 is shared between the Kelani and Mahaweli. The *rag1* network of *Plesiopuntius bimaculatus* consists of a total of six haplotypes (Fig. 6d). H1 represents subclade 1. However, the phylogeographic structure observed in subclades 2 and 3 of the mitochondrial haplotypes is not apparent in *rag1*. H6 appears to be a widespread haplotype, present in individuals representing both subclade 2 and subclade 3, while H5 is shared between headwaters of the Gal and Mahaweli.

**Figure 7:**
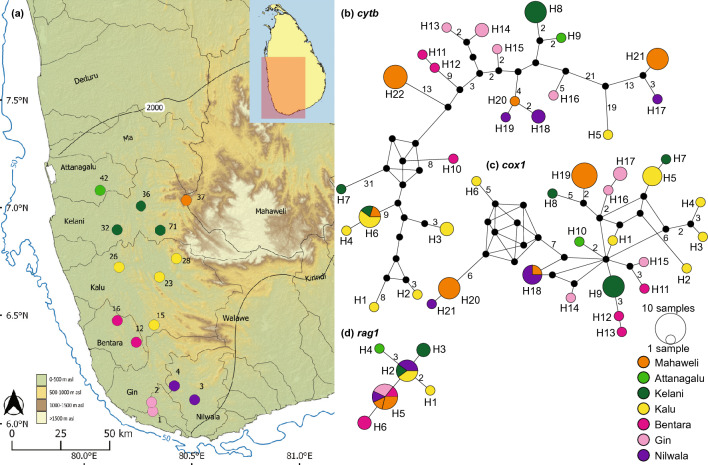
(**a**) Indication of the geographical origin of samples of *Rohanella titteya* used in the molecular analysis. Numbers on the map represent the sampling localities listed in Supplementary Table S3. Median-joining haplotype networks for *R. titteya* based on the analysis of (**b**) a 1073 bp fragment of the *cytb* gene, (**c**) a 630 bp fragment of the *cox1* gene, and (**d**) a 1470 bp fragment of the *rag1* gene. The number of mutational steps > 1 is shown. The black circles are hypothetical nodes. Legend colors correspond to river basins. The bold black line in Sri Lanka indicates the 2000-mm isohyet, which encompasses the wet zone. The 50 m isobath (dark blue) is based on GEBCO Compilation Group (2020). Major river basins in Sri Lanka are labelled in black.

*Rohanella titteya* (Fig. 7) has a network containing 22 *cytb* haplotypes, of which all except H6 are confined to individual basins (Fig. 7b). H6 is shared between the contiguous Kelani and Kalu basins, and represented in some translocated populations in the Mahaweli basin as well. Haplotypes H20-H22, which also occur in translocated populations in the Mahaweli basin, are apparently unique. A clear phylogeographic structure in the median-joining haplotype network is, however, not apparent. The *cox1* network contains 21 haplotypes; it is similar to the *cytb* network, with no evident phylogeographic structure (Fig. 7c). All except H18 are confined to individual basins. H18 is confined to the Nilwala basin, though present also in some individuals of a translocated population in the Mahaweli. The *rag1* network contains six haplotypes, of which H1, H3, H4, and H6 are unique to the Kalu, Kelani, Attanagalu, and Bentara basins, respectively (Fig. 7d). H2 is common to the Nilwala, Kalu, and Kelani basins, while H5 is common to the Bentara, Gin and Nilwala, though present also in translocated populations in the Mahaweli.

**Figure 8:**
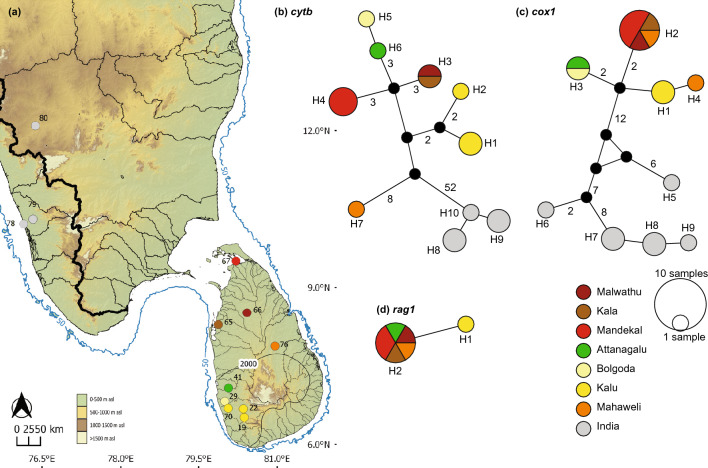
(**a**) Indication of the geographical origin of samples of *Bhava vittata* used in the molecular analysis. Numbers on the map represent the sampling localities listed in Supplementary Table S3. Median-joining haplotype networks for *B. vittata* based on the analysis of (**b**) a 1073 bp fragment of the *cytb* gene, (**c**) a 630 bp fragment of the *cox1* gene, and (**d**) a 1470 bp fragment of the *rag1* gene. The number of mutational steps > 1 is shown. The black circles are hypothetical nodes. Legend colors correspond to river basins. The bold black line in Sri Lanka indicates the 2000-mm isohyet, which encompasses the wet zone. The bold black line along the axis of the Western Ghats indicates the boundary between east- and west-flowing drainages. The 50 m isobath (dark blue) is based on GEBCO Compilation Group (2020). Major river basins in Sri Lanka are labelled in black.

*Bhava vittata* (Fig. 8) has a *cytb* network formed of 10 haplotypes (Fig. 8b). Seven of these (H1-H7) are confined to Sri Lanka, while H8-H10 are restricted to southern India. None are shared between India and Sri Lanka. H3 is shared between the contiguous Kala and Malwathu basins of the northern dry zone, while the rest of the haplotypes are confined to individual basins. Nevertheless, a clear *cytb* phylogeographic structure is not apparent within Sri Lanka. The *cox1* network of *Bhava vittata* contains 9 haplotypes (Fig. 8c). Four (H1-H4) of these are confined to Sri Lanka, while H5-H9 are confined to southern India, with none shared between the two. A clear phylogeographic structure, however, is not apparent for *cox1*. The *rag1* network contains only two haplotypes, of which H2 appears to be a widespread, present in both climatic zones of the island (Fig. 8d).

## Discussion

### General biogeography

Through a review of all animal taxa for which data were available, Pethiyagoda & Sudasinghe^1^ presented a tentative historical scenario for Sri Lanka’s freshwater fishes, summarized as follows. Sri Lanka’s fish fauna is derived entirely from colonists from India, which in turn entered India subsequent to the India-Asia collision ~ 52 Ma. The colonization of Sri Lanka was contingent on the Palk Isthmus being subaerial and its climate being conducive to the dispersal of freshwater fishes. Both these conditions obtained only briefly and intermittently since the early Miocene. As a result, lineages associated with a perhumid climate, such as the endemic diversification of *Systomus* (*S. asoka* (Kottelat & Pethiyagoda)*, S. martenstyni* (Kottelat & Pethiyagoda) and *S. pleurotaenia* (Bleeker)), *Rasboroides-Horadandia* (Cyrprinidae), *Belontia*, and *Malpulutta-Pseudosphromenus* (Osphronemidae), appear to have colonized Sri Lanka only between the early Oligocene eustatic sea-level depression and the inundation of the Palk Isthmus during the Burdigalian Miocene^9,14–17^. Although the Isthmus was emergent for most of the time from 14 Ma until it was finally submerged in the early Holocene, its climate seems to have been inconducive to the dispersal of wet-adapted species. Indeed, instances of even dry-adapted taxa crossing the Isthmus during the Plio-Pleistocene are rare. During this period, presumably owing to an arid climate, the land bridge served more as a biotic filter than a corridor^1^. Our results are consistent with such a scenario.

Except for the P. sophore group, which is distributed also in the Ganges basin and to the east of IBR in Southeast Asia, the diversity of *Puntius* s.l. is confined to the Indian peninsula and Sri Lanka^11^. It appears therefore, that this group originated and diversified in this region, with subsequent dispersal via the Ganges basin to Southeast Asia as evidenced by our divergence timing and biogeographic analyses. The biogeography of the P. sophore group further supports this ‘out-of-India’ to Southeast Asia hypothesis^18–20^.

*Rohanella* + *Plesiopuntius* diverged from *Puntius*, its sister group, 17.3 (95% HPD 14.8–20.0) Ma. However, given convincing geological and paleobiogeographic evidence that the Palk Isthmus was inundated 17–14 Ma^1,15^, it appears likely that the common ancestor of *Rohanella* and *Plesiopuntius* colonized Sri Lanka between 20 and 17 Ma. This is consonant with the 22.4 (95% HPD 16.6–27.9) Ma crown age for the endemic clade of *Systomus* recovered by Sudasinghe et al.^9^. As seen from our biogeographic analysis, the basal ancestors of *Puntius* s.l. appear to be widespread generalists, including the common ancestor of *Plesiopuntius* + *Rohanella* which, having crossed the Palk Isthmus into Sri Lanka, went on to diversify in the perhumid southwest.

With few exceptions such as *P. kamalika*, the freshwater fishes of Sri Lanka’s north-western dry zone occur also in India. These generalist members of *Puntius* s.l. in Sri Lanka, such as *Puntius kamalika, P. thermalis* and *Bhava vittata*, occur largely in open (unshaded), swamp-like habitats. Based on this observation, Pethiyagoda & Sudasinghe^1^ hypothesized that *P. thermalis*, hitherto considered a Sri Lankan endemic, is likely not one. Our results show this to be the case. The species is a generalist of the lowland floodplains and lacks strong phylogeographic structure. Although the availability of only a single Indian sample makes a definitive conclusion impossible, it is likely that *P. thermalis* colonized Sri Lanka only recently. The two independent dispersal events out of India (India + Ganges Basin) to Southeast Asia within the P. sophore group, which are lowland generalists, is plausible given the evidence for dispersal of lowland swamp elements of palynoflora between India and Southeast Asia since the beginning of the Miocene^21^.

Unusually for a fish endemic to Sri Lanka, *P. kamalika* occurs throughout the island’s north-western lowlands, up to an elevation of about 65 m asl^1^. It too, lacks clear phylogeographic structure. Unlike other widely distributed species of *Puntius*, *P. kamalika* is nowhere abundant, usually occurring in small groups. The lack of samples from India precludes a conclusion as to whether it occurs also on the subcontinent and colonized Sri Lanka from there. The present evidence, however, suggests an instance of vicariance between the common ancestor of *P. kamalika* and the southern Indian *P. mahecola* during the Plio-Pleistocene (Fig. 1).

*Bhava vittata* too, is a generalist which occurs in the lowland floodplains (< 600 m asl) of all Sri Lanka’s climatic zones. It too, lacks clear phylogeographic structure, though the Sri Lankan population appears to be genetically distinct from that in southwestern India (no samples were available from Southeast India).

The lack of phylogeographic structure in Sri Lanka in *Puntius thermalis*, *P. kamalika*, and *Bhava vittata* resembles the pattern observed in other generalist freshwater fishes such as *Channa kelaartii* (Günther)*, Devario malabaricus, Rasbora dandia* (Valenciennes) and *Dawkinsia filamentosa* (Valenciennes)*,* which are shared between the island and the mainland^7,8,10,22^. All are widespread generalists and there appear to be no barriers to their dispersal between basins across the littoral floodplain.

In contrast to the preceding three species, *P. dorsalis*, a low-mid elevation species (< 765 m asl) and *Plesiopuntius bimaculatus,* which is widely distributed across all of Sri Lanka’s climatic zones (< 1230 m asl), exhibit strong phylogeographic structure. Each species is composed of three subclades. The common ancestor of *P. dorsalis* and the south-western endemic *P. kelumi* appears to have split in Sri Lanka in the late Miocene (9.4 Ma, 95% HPD 7.7–11.2 Ma), while that of *Plesiopuntius bimaculatus* and *Rohanella titteya* appears to have split in Sri Lanka in the mid-Miocene (13.1 Ma, 95% HPD 10.7–15.7 Ma).

We note two color morphs in *P. dorsalis*: a golden one, occurring primarily in the wet zone, northwestern dry zone and the highland Knuckles region of the Mahaweli basin (subclade 2); and a silvery one inhabiting the wet-zone and Mahaweli (except the Knuckles hills), and the northern and eastern dry and intermediate zones (subclades 1 and 3). Based on field observations, the golden form (subclade 2) appears to be restricted to riverine habitats. Subclade 3, meanwhile, is a generalist found in both lotic and lentic environments. We suspect that more extensive future sampling may reveal an ecological differentiation between these subclades.

In contrast to the preceding species, *P. kelumi* and *Rohanella titteya* are confined to the island’s perhumid southwest, with upper elevation limits of 380 m and 870 m, respectively^1^. *Puntius kelumi* occurs as three subclades: 1, from the southern Gin and Nilwala basins; 2, ranging widely, from the Attanagalu to the Gin basins; and 3, apparently confined to the Kalu basin. The species exhibits strong phylogeographic structure, with haplotypes shared between even adjacent basins only rarely, a pattern observed also in other southwest endemics such as *Devario micronema* (Bleeker)*, Laubuka varuna* (Pethiyagoda, Kottelat, Silva, Maduwage & Meegaskumbura)*, Systomus pleurotaenia* (Bleeker)*,* and *Pethia nigrofasciata* (Günther)^8,9,23,24^. The representation of both subclades 1 and 2 in the Gin basin may suggest two independent colonizations of that basin, as observed also in *P. nigrofasciata*^24^.

### Rainforest refugia

Ashton^12^ suggested that when the intensity of the monsoons abated during glacial maxima, Sri Lanka’s evergreen rain forests retreated to refugia in moist valleys, with diurnal cloud-shadow helping to ameliorate desiccation. Proximity to the ocean and onshore winds too, deliver orographic rainfall, helping to maintain such refugia. Farooqui et al.^25^ inferred such a scenario also in the Western Ghats.

*Plesiopuntius bimaculatus,* which is widely distributed across Sri Lanka’s climatic zones to an elevation of about 1230 m asl, comprises of three subclades. Subclade 1, which is the sister group of subclades 2 + 3, is associated with rainforests. This suggests that the Kalu-Gin-Nilwala basins, which drain the island’s perhumid southwest, served as a refuge for fishes. This pattern is repeated also in the case of other cyprinid lineages confined to south-western drainages, such as *Puntius kelumi*, *Systomus pleurotaenia*, and the micronema clade of *Devario*, in which lineages confined to the Gin and Nilwala basins of the perhumid zone show a sister-group relationship to populations in the more seasonal northerly basins^present study^^8,9^.

Similarly, the basal group of *Puntius dorsalis* is confined to the headwaters of southeastern Walawe and Menik basins, reflecting the phylogeographic patterns observed also for *Garra ceylonensis* Bleeker*.* This suggests that the headwaters of the island’s south-eastern rivers, such as the Walawe, Menik, and Gal once served as drought refugia^1,26^. This is evidenced also by their harboring greater genetic diversity even now. In the case of *P. dorsalis* and *Pl. bimaculatus*, it appears that the island was recolonized when pluvial conditions resumed. Indeed, the lineage diversity within *Puntius* in Sri Lanka’s perhumid southwest is higher than elsewhere in the island, suggestive of a species pump.

*Rohanella titteya* is unique among the nine cyprinid species confined to Sri Lanka’s perhumid south-western region in exhibiting no clear phylogeographic structure. The star-like haplotype networks of this species (Fig. 7), along with the low nucleotide diversity, high haplotype diversity, and negative Tajima’s D (though not significant) for all gene markers included here may suggest that this exclusive rainforest associate has undergone a recent range expansion following a bottleneck, presumably having survived Pleistocene desiccation in a perhumid refugium.

Relictual narrow-range endemics occur also at some other perhumid locations in the hills and foothills, such as Aranayake in the Maha basin (*Devario memorialis* Sudasinghe, Pethiyagoda & Meegaskumbura), the headwaters of the Gal basin (*Laubuka hema* Sudasinghe, Pethiyagoda & Meegaskumbura), Bopattalawa in the upper Mahaweli basin (*Devario monticola* Batuwita, de Silva & Udugampala), and Weralugahamula in the Kalu basin for *Rasbora armitagei* Silva, Maduwage & Pethiyagoda^8,10,23^*.* Interestingly, these authors found populations confined to refugia to have greater genetic diversity, as indeed do we in the case, for example, of the population of *Puntius dorsalis* at Aranayake in the Maha basin, which is separated from other conspecifics by a minimum 80 and 26 mutational steps in *cytb* and *cox1*, respectively, but contains the same *rag1* haplotype as specimens from the contiguous Attanagalu and Kelani rivers.

The identification of these hitherto unidentified refugia paves the way for their more intensive exploration targeting other faunal and floral groups which too, may harbor previously unsuspected diversity.

### ‘Out of Sri Lanka’

On account of its remarkable endemism and species richness, the perhumid region of southwestern Sri Lanka is thought of as a floristic refugium^12^. This region exhibits the least seasonality in rainfall anywhere in South Asia, resembling instead the ever-wet equatorial climate of the Malesia^1^. The persistence in this region of ‘Gondwanan’ plant taxa such as *Axinandra* and *Trichadenia*, which are dependent on a perhumid climate and are absent from India, has been taken as evidence of its refugial status^27^.

Our results offer the first evidence that freshwater fishes too, may have survived periods of inter-pluvial desiccation in such refugia. The basal group of the widely distributed *P. dorsalis*, for example, is confined to the headwaters of south-eastern basins. It appears that *P. dorsalis* evolved in Sri Lanka and went on to colonize India. Similarly, the basal subclade of *Plesiopuntius bimaculatus* too, is confined to the perhumid southwest, with the dry-zone clade (subclade 3) having gone on to colonize India (Fig. 1). It is noteworthy that despite *Plesiopuntius bimaculatus* having a much larger range in India than in Sri Lanka (Fig. 6a), the Indian population shows low genetic diversity and scant evidence of diversification, perhaps suggestive of rapid colonization following a recent migration event.

The species of *Puntius* s.l. that are shared between India and Sri Lanka appear to have crossed the Palk Isthmus during the Plio-Pleistocene (Fig. 1, Table 1): *P. thermalis* and *B. vittata* from India to Sri Lanka, and *P. dorsalis* and *Pl. bimaculatus* from Sri Lanka to India. Evidence for Sri Lanka-India back-migrations from other faunal groups, however, is scarce: the case of *Lepidocephalichthys thermalis* been the only other known case for freshwater fishes^28^. Beenaerts et al.^6^ and Meegaskumbura et al.^5^ showed, however, that freshwater crabs and shrub frogs too, had on occasion gone on to colonize India from Sri Lanka. This evidence adds credence to the speculation by Bose et al.^13^ that the close phylogenetic relatedness of rainforest endemics between the southern Western Ghats and Sri Lanka was perhaps a result of these taxa having persisted in Pleistocene refugia in the island, going on to disperse to the Western Ghats via the Palk Isthmus, and then undergoing parapatric speciation in India.

### Highland extinction

Sri Lanka is unusual in that its lowland rainforest flora shows its closest affinity to that of Malesia, whereas that of the highlands, which rise to 2524 m asl, is clearly derived from the flora of the Western Ghats mountains of southern India. This led Pethiyagoda & Sudasinghe^1^ to posit a prior climate-driven biotic extinction in the highlands. By way of support for this hypothesis, these authors pointed to the paucity of fishes in the highlands. Only two species, *Garra ceylonensis* and *Devario monticola*, occur at an elevation above 1500 m asl, both as isolated, presumably relictual populations. The remaining fishes in the highlands, such as *Devario malabaricus*, are species that occur in the northern dry zone and colonized the montane region only recently^8^. The present results too, corroborate such a scenario. The only species of *Puntius* s.l. present in the montane zone, up to an elevation of 1230 m asl, is *Plesiopuntius bimaculatus*^1^. This montane population belongs to subclade 3, whose range extends to the northern Malwathu and Kala basins, both in the dry zone, and also southern India, suggestive of recent highland colonization. The phylogeographic patterns of the only other freshwater fish species that is recorded above 1300 m asl in Sri Lanka, the nemacheilid loach genus *Schistura*, remains to be investigated. However, we hypothesize a recent highland colonization in this case, too.

### Headwater capture

Several recent studies in Sri Lanka have found evidence of freshwater fish geneflow across upland watersheds as a result of presumed headwater capture events: e.g., in *Systomus pleurotaenia* and *Dawkinsia srilankensis*^7,9^. The present data too, offer evidence of dispersal across watersheds which lack connectivity via a lowland floodplain. Subclade 3 of the Mahaweli population of *Plesiopuntius bimaculatus*, for example, shares the H23 *cytb* haplotype with the Attanagalu-Deduru-Ma basins to the west, and the H12 *cytb* haplotype in subclade 2 with the Gal basin to the east. In the case of *Laubuka hema* and *Systomus sarana* too, it is evident that there has been trans-basin gene flow between the uplands of the Mahaweli and Gal drainages^9,23^. Likewise, in the case of *Garra ceylonensis*, Sudasinghe et al.^26^ showed there to have been trans-basin gene flow between the uplands of the Deduru-Ma-Attanagalu-Kelani basins, while in the case of *Channa orientalis* Bloch & Schneider, there has been trans-basin gene flow between the uplands of the Kelani and Mahaweli basins^29^.

The disjunct distribution of subclade 2, the golden form of *P. dorsalis* between the Knuckles hills of Mahaweli and southwestern basins too, suggests an historical river capture. Sudasinghe et al.^7^ argued that the Kala basin, in which subclade 2 of *P. dorsalis* too occurs, may have historically drained part of the Knuckles Hills. Similarly, the distribution of the endemic snakehead *Channa orientalis* in the Mahaweli and Kelani basins appears to have resulted from an historical upland river-capture event^29^.

Likewise, the occurrence in the Gin basin of *Puntius kelumi* representative of two different clades (Fig. 6b,c) may be suggestive of recent colonization from adjacent basins. This structure has previously been shown to occur in the southern Gin and Nilwala basins also in the case of other cyprinid species such as *Garra ceylonensis, Systomus pleurotaenia* and *Pethia nigrofasciata*^9,24,26^.

Masuda et al.^30^ showed that in highly dynamic areas surrounded by active faults, stream capture may be more frequent, aiding the inter-drainage dispersal of freshwater organisms. Our results suggest that such stream capture events seem to have been frequent in Sri Lanka despite the island’s tectonic stability since the Precambrian^31^. The perhumid zone’s intense rainfall, however, may have exacerbated the intensity and frequency of landslides^32^, precipitating capture events.

## Conclusions

Our results support the emerging framework of Sri Lankan biogeography^1^. Different lineages of *Puntius* s.l. diversified on Sri Lanka at different times, the earliest being the common ancestor of *Plesiopuntius-Rohanella* in the early Miocene. Rainforest refugia in the island’s southwest acted as species pumps, facilitating albeit modest diversifications of taxa that went on to colonize the island while dispersing also to India whenever a wet climate prevailed and when the Palk Isthmus was emergent. The single species of *Puntius* s.l. present in the highlands is a recent colonizer, corroborating earlier studies which suggested a prior extirpation of the montane ichthyofauna. There is substantial evidence of dispersal across watersheds through headwater stream capture. We urge further exploration of the refugia we identify because these may contain other taxa that survived previous desiccation events. The increasing seasonality of rainfall resulting from climate change greatly increases the utility of rainforest refugia as foci for conservation attention, making it imperative that these habitats be demarcated, monitored and conserved.

## Methods

### Ethics declaration

Fieldwork was carried out in Sri Lanka under the permissions and guidelines from the Department of Wildlife Conservation and the Forest Department (permit no. WL/3/2/59/14 and R&E/RES/NFSRCM/14-16-4, respectively). Sampling in India was carried out under the permits issued by the Maharashtra State Biodiversity Board to ND and Kerala State Forest and Wildlife Department to RR. The ethical committee of the Postgraduate Institute of Science, University of Peradeniya, approved the methods of specimen collection, euthanization (using tricaine methanesulfonate), tissue sampling and specimen fixation at its 27th meeting held on 4 August 2017.

### Geography

The topography of Sri Lanka (65,610 km^2^) is characterized by the central mountains, which rise to 2524 m. Some 103 rivers drain the island, the larger among which radiate from the central mountains. Rainfall is characterized by two monsoons: the central mountains, and the island’s southwest quarter generally, benefit from both. This ‘wet zone’ receives an annual rainfall > 2 m. Within the wet zone lies a ‘perhumid zone’ in which average monthly precipitation remains above 100 mm even during the dry season, which lasts three or fewer months^1^. This perhumid climate supports mixed-dipterocarp rainforests in the lowlands and tropical montane cloud forests in the upper montane region. The remaining two-thirds of the island is a ‘dry zone’ (rainfall < 2 m/y), with the ‘arid zones’ of the northwest and southeast littoral receiving just 0.5–1.5 m of rain per year. Most of the island’s endemic fishes are restricted to the wet zone, preponderantly to the shallow, heavily shaded streams draining the rainforests of the perhumid zone^1^.

### Definitions

For reasons of brevity, we use ‘India’ to mean greater Peninsular India, approximately that part of the subcontinent south of the 25°N parallel. We label species associated with clearwater rainforest streams and rivers as habitat specialists, and those associated with unshaded, lowland lentic or lotic waters (large rivers, wetlands), whether in the wet zone or the dry zone, as habitat generalists. In the text, clades treated as species groups carry the name of the oldest included species, but in Roman characters, to avoid confusion with italicized Linnaean binomials. We apply the term *Puntius* s.l. to the group of fishes that includes *Puntius *sensu stricto (the P. sophore, P. mahecola, and P. dorsalis species groups of Sudasinghe et al.^11^ as well as *Plesiopuntius bimaculatus*, *Rohanella titteya*, and *Bhava vittata*.

### DNA protocols

Gene nomenclature follows the ZFIN Zebrafish Nomenclature Conventions (https://goo.gl/MdawKQ). The molecular phylogeny of Sudasinghe et al.^11^ generated 145 mitochondrial cytochrome b (*cytb*), 152 mitochondrial cytochrome c oxidase subunit 1 (*cox1*), 95 nuclear recombination activating protein 1 (*rag1*), and 35 interphotoreceptor retinoid-binding protein (*irbp*) sequences representative of 153 individuals of *Puntius* s.l. sampled at 67 locations in Sri Lanka. In the present study, we use primarily this dataset, but in addition generated new 24 *cytb*, and 5 *cox1* sequences for representatives of Indian *Puntius* s.l. (Supplementary Table S2). Methods of DNA extraction, PCR amplification and PCR product purification for *cytb* and *cox1* of Indian samples follow^33^. ChromasPro v1.34 (Technelysium Pty Ltd, Australia) and MEGA v. 7.0^34^ were used to check the newly generated sequences and construct the consensus sequences of the 5′ and 3′ strands, respectively.

### Phylogenetic analysis

Phylogenetic inference for the mitochondrial + nuclear (*cytb* + *cox1* + *rag1* + *irbp*), hereafter (combined) dataset (3964 bp, 413 taxa) was carried out based on Maximum Likelihood (ML) inference using RAxML-NG^35^. The optimal nucleotide substitution model for the dataset was determined using ModelTest-NG v0.1.7^36^, with each codon position of each gene given as the starting subset, and model selected based on the Akaike Information Criterion (AIC). Felsenstein bootstrapping for 1000 replicates was carried out in RaxML-NG to determine the statistical support for the nodes in the ML tree.

### Divergence time estimation

The interspecific divergence timings within *Puntius* s.l. were estimated based on the combined dataset in BEAST 2^37^. We selected an individual representing each lineage as a Molecular Operational Taxonomic Unit (MOTU)^38^ following Sudasinghe et al.^11^, resulting in a 22-taxon dataset for the divergence estimation analysis. The substitution model was determined using ModelTest-NG v0.1.7, providing a single partition per gene in accounting for over-parameterization. We used a relaxed clock under lognormal distribution as the clock prior, and a Yule pure-birth model as the tree prior. Given the narrow taxonomic focus of our study, and noting also that many of the deeper phylogenetic relationships within Cypriniformes remain poorly resolved^39^, we chose not to use fossil calibration. Further, we omitted all taxa outside *Puntius* s.l. from the analysis. Instead, we used the average cyprinid *cytb* substitution rate of 0.0082 substitutions per site per million years, with a normal distribution, to calibrate the *cytb* clock rate^17,40^. The *cox1*, *rag1*, and *irbp* substitution rates were estimated relative to *cytb*. This substitution rate for *cytb* has been derived in reference to European cyprinids, based on reliably dated geological events^41^.

RAxML-NG and BEAST analyses were performed on UBELIX (http://www.id.unibe.ch/hpc), the HPC cluster at the University of Bern, Switzerland. The trees obtained from ML and BEAST analyses were visualized using Figtree v1.4.3 (http://tree.bio.ed.ac.uk/software/figtree).

### Ancestral-range reconstruction

To explore the biogeographic history of *Puntius* s.l., we estimated ancestral distributions using the R-package BioGeoBEARS^42^, using the dispersal–extinction–cladogenesis (DEC) model^43^ and DEC + J model, and the best-fit model was assessed using the AICc. The maximum clade credibility (MCC) tree from the BEAST analysis, after removing the outgroup, was used as the input tree for the biogeographic analysis. We considered a total of four areas: Sri Lanka (A), India (B), Ganges Basin (C), and Southeast Asia (D) for the biogeographic analysis. The maximum number of areas of ancestral ranges was specified as two, which is the maximum number of unit ranges for the most widely distributed species in the dataset. We did not impose any dispersal constraints. In the case of *Puntius dorsalis*, sequences from India were not available. As hypothesized by Sudasinghe et al.^11^, we considered subclade 3 of *P. dorsalis* to be shared between Sri Lanka and India. This is the most widespread subclade of *P. dorsalis* in Sri Lanka, extending to the northern dry zone at its closest proximity to India. This is supported by previous phylogeographic studies that show that cyprinid species in other genera such as *Dawkinsia filamentosa, Systomus sarana* (Hamilton)*, Rasbora microcephala* (Jerdon)*, Devario malabaricus* (Jerdon)*,* and *Esomus thermoicos* (Valenciennes)*,* whose range extends to the northern dry zone, are shared with southern India^7–10,44^.

### Genetic diversity and phylogeography

The haplotype networks for *cytb*, *cox1*, and *rag1* for Sri Lankan members of *Puntius* s.l. were reconstructed through a median-joining network^45^ in PopArt^46^. For each of these genes, we also estimated genetic diversity by computing the number of haplotypes (h), polymorphic sites (S), parsimony-informative sites (P), nucleotide diversities (π), and haplotype diversities (Hd), using DnaSP v.6^47^. The neutrality tests, Tajima’s D^48^ and Fu and Li’s F^49^, were carried out in DnaSP v.6 to explore demographic changes. Because only a few individuals of each species were sequenced for *irbp*, haplotype networks and genetic diversity were not computed for this molecular marker.

### Supplementary Information

Supplementary Information.

## Data Availability

The DNA sequences generated and analyzed in this study are openly available in the GenBank http://www.ncbi.nlm.nih.gov/genbank and their accession numbers are provided in Supplementary Tables S2 and S3.
