# Supplementary material for: Biogeography and evolutionary history of Puntius sensu lato (Teleostei: Cyprinidae) in Sri Lanka
Source: Sci Rep. 2023 Oct 31;13:18724. doi: 10.1038/s41598-023-45377-9 (PMC10618540; doi:10.1038/s41598-023-45377-9)
Supplement: Supplementary file 1 — Supplementary Information. [file 41598_2023_45377_MOESM1_ESM.pdf]

Research Article

Biogeography and evolutionary history of *Puntius* sensu lato (Teleostei: Cyprinidae) in Sri Lanka

Running title: *Puntius* sensu lato biogeography

Hiranya Sudasinghe[1,2,3,4], Tharindu Ranasinghe[5], Neelesh Dahanukar[6], Rajeev Raghavan[7], Lukas Rüber[4,8], Rohan Pethiyagoda[9], Madhava Meegaskumbura[10,*]

[1]Evolutionary Ecology and Systematics Laboratory, Department of Molecular Biology and Biotechnology, University of Peradeniya, Peradeniya, 20400, Sri Lanka
[2]Postgraduate Institute of Science, University of Peradeniya, Peradeniya, 20400, Sri Lanka
[3]Evolutionary Ecology, Institute of Ecology and Evolution, University of Bern, 3012 Bern, Switzerland
[4]Naturhistorisches Museum Bern, Bernastrasse, 15, 3005 Bern, Switzerland
[5]Wild Island Foundation, 6A, Mendis Lane, Moratuwa, 10400, Sri Lanka
[6]Department of Life Sciences, School of Natural Sciences, Shiv Nadar Institution of Eminence, Delhi-NCR, India
[7]Department of Fisheries Resource Management, Kerala University of Fisheries and Ocean Studies (KUFOS), Kochi, India
[8]Aquatic Ecology and Evolution, Institute of Ecology and Evolution, University of Bern, 3012 Bern, Switzerland
[9]Ichthyology Section, Australian Museum, 1 William Street, Sydney, NSW 2010, Australia
[8]Guangxi Key Laboratory for Forest Ecology and Conservation, College of Forestry, Guangxi University, Nanning, 530004, Guangxi, People's Republic of China
[*]Correspondence: madhava_m@mac.com

Supplementary material

Supplementary Fig. S1. Molecular phylogenetic relationships of Similiogastrinae, with a focus on *Puntius sensu lato*, based on Maximum likelihood inference of the concatenated mitochondrial + nuclear (3964 bp, 413 taxa) dataset. Node support represent ML bootstrap and values below 70 is not shown. Scale bar represents number of changes per site. Numbers in square brackets represent the subclades in different species groups of *Puntius* s.l. following Sudasinghe et al. (2023). The double solidus (slash) at the outgroup indicates that it is not to scale.

Supplementary Table S1. Genetic diversity, based on *cytb*, *cox1* and *rag1*, in Sri Lankan species of *Puntius sensu lato*.
Number of sequences (N), number of haplotypes (h), polymorphic sites (S), parsimony-informative sites (P), nucleotide diversity (π), haplotype diversity (Hd). None of the neutrality tests was statistically significant.

Supplementary Table S2. Specimens of Indian species from which sequences were generated for the molecular analyses in the present study, with their localities, voucher references, and GenBank accession numbers.

Supplementary Table S3. Details of the sequences of Sri Lankan and Indian *Puntius* s.l. used in the haplotype network analyses with their localities, voucher references, and GenBank accession numbers.

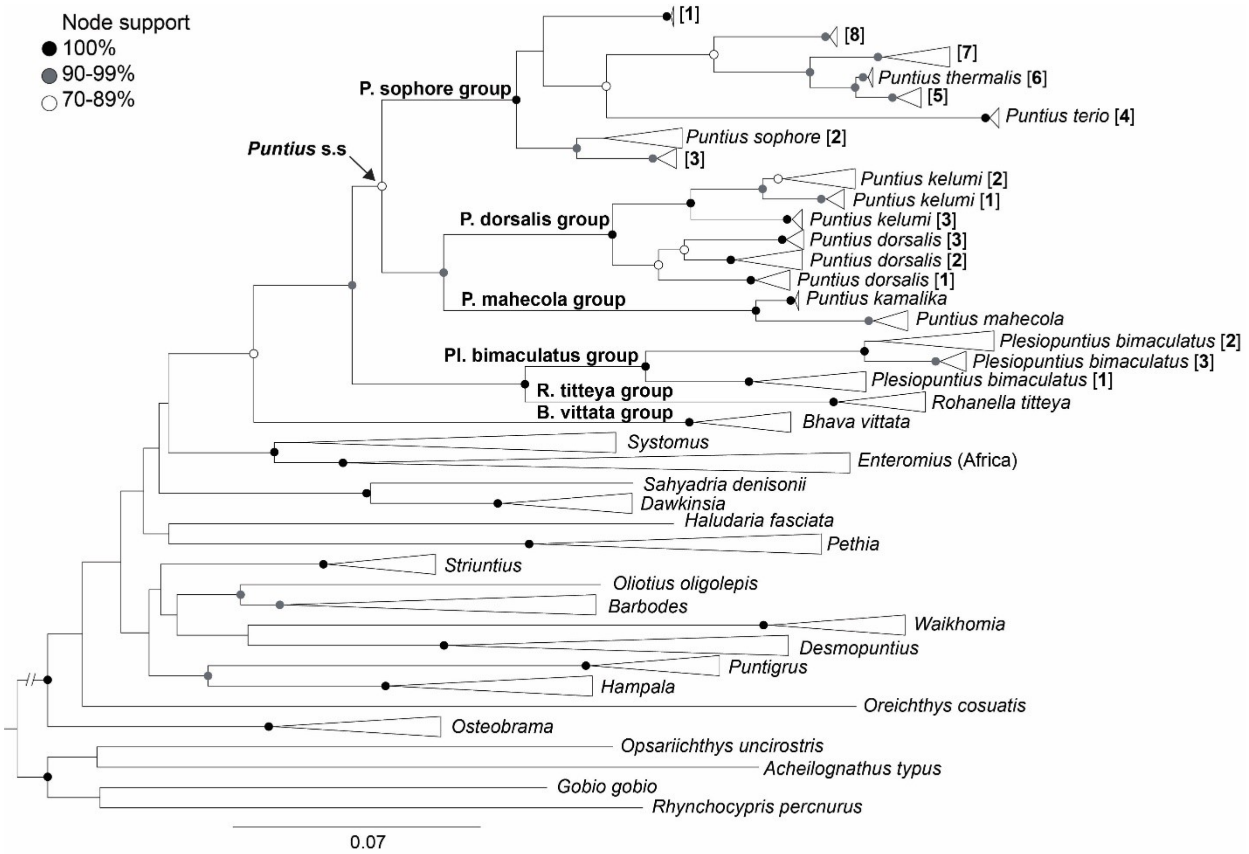

Supplementary Fig. S1. Molecular phylogenetic relationships of Similiogastrinae, with a focus on *Puntius sensu lato*, based on Maximum likelihood inference of the concatenated mitochondrial + nuclear (3964 bp, 413 taxa) dataset. Node support represent ML bootstrap and values below 70 is not shown. Scale bar represents number of changes per site. Numbers in square brackets represent the subclades in different species groups of *Puntius* s.l. following Sudasinghe et al. (2023). The double solidus (slash) at the outgroup indicates that it is not to scale.

Supplementary Table S1. Genetic diversity, based on *cytb*, *cox1* and *rag1*, in Sri Lankan species of *Puntius sensu lato*. Number of sequences (N), number of haplotypes (h), polymorphic sites (S), parsimony-informative sites (P), nucleotide diversity (π), haplotype diversity (Hd). None of the neutrality tests was statistically significant.

**Species**	**Gene**	**N**	**H**	**S**	**P**	**π**	**Hd**	**Tajima's D test**	**Fu and Li's F test**
Puntius thermalis	*cytb*	16	8	9	5	0.0019	0.875	-0.8178	-0.7009
	cox1	20	4	4	1	0.0023	0.711	0.6862	0.3641
	rag1	12	1	0	0	-	-	-	-
P. kamalika	*cytb*	7	4	4	3	0.0016	0.714	0.5178	0.6738
	cox1	7	1	0	0	-	-	-	-
	rag1	6	1	0	0	-	-	-	-
P. dorsalis	*cytb*	28	17	219	178	0.0615	0.963	0.4426	0.5102
	cox1	30	14	92	78	0.0460	0.920	0.6359	0.6861
	rag1	22	6	13	12	0.0030	0.727	0.8135	1.1711
P. kelumi	*cytb*	10	7	115	77	0.0474	0.911	0.8094	0.3913
	cox1	15	12	84	79	0.0550	0.971	1.3152	1.5366
	rag1	10	3	2	1	0.0005	0.644	0.1203	-0.2046
Plesiopuntius bimaculatus	*cytb*	48	32	283	270	0.0685	0.977	0.1105	1.2640
	cox1	44	18	119	115	0.0515	0.934	0.0836	1.4243
	rag1	26	6	7	5	0.0028	0.517	-0.9713	-0.3843
Rohanella titteya	*cytb*	42	22	137	88	0.0239	0.944	-0.7864	-1.2557
	cox1	42	21	54	33	0.0148	0.937	-1.1549	-1.5960
	rag1	18	6	8	3	0.0010	0.784	-1.2292	-1.5507
Bhava vittata	*cytb*	16	10	76	66	0.0274	0.942	1.2273	1.2373
	cox1	18	9	42	32	0.0251	0.876	0.9677	0.5615
	rag1	7	2	1	0	0.0002	0.286	-1.0062	-1.1014

Supplementary Table S2. Specimens of Indian species from which sequences were generated for the molecular analyses in the present study, with their localities, voucher references, and GenBank accession numbers.

Voucher	Genus	Species	Location	Latitude	Longitude	Locality	*cytb*	*cytb* haplotype	*cox1*	*cox1* haplotype	*rag1*	*rag1 haplotype*	*irbp*	Subclade
PuSuCu1	*Puntius*	*thermalis*	India: Cuddalore, Alappakkam	11.75 N	79.708 E	88	OR667729	H8	NA	NA	NA	NA	NA	Sophore_6
PuBB1	*Plesiopuntius*	*bimaculatus*	India: Nelliyampathy, Bharathapuzha	10.552 N	76.717 E	81	OR667730	H32	OR666946	H18	NA	NA	NA	Bimac_3
PuBB2	*Plesiopuntius*	*bimaculatus*	India: Nelliyampathy, Bharathapuzha	10.552 N	76.717 E	81	OR667731	H32	OR666947	H18	NA	NA	NA	Bimac_3
PuBC1	*Plesiopuntius*	*bimaculatus*	India: Nelliyampathy, Chalakudy	10.538 N	76.677 E	82	OR667732	H32	OR666948	H18	NA	NA	NA	Bimac_3
PuBiC1	*Plesiopuntius*	*bimaculatus*	India: Chuchi falls	12.351 N	77.447 E	83	OR667733	H28	NA	NA	NA	NA	NA	Bimac_3
PuBiC2	*Plesiopuntius*	*bimaculatus*	India: Chuchi falls	12.351 N	77.447 E	83	OR667734	H28	NA	NA	NA	NA	NA	Bimac_3
PubiE1	*Plesiopuntius*	*bimaculatus*	India: Eastern ghats	18.326 N	82.873 E	84	OR667735	H27	NA	NA	NA	NA	NA	Bimac_3
PuBiKa1	*Plesiopuntius*	*bimaculatus*	India: Kamra	15.372 N	74.413 E	85	OR667736	H30	NA	NA	NA	NA	NA	Bimac_3
PuBiS2	*Plesiopuntius*	*bimaculatus*	India: Shimoga	13.87 N	75.564 E	86	OR667737	H29	NA	NA	NA	NA	NA	Bimac_3
PuBiY1	*Plesiopuntius*	*bimaculatus*	India: Yercaurd	11.783 N	78.21 E	87	OR667738	H31	NA	NA	NA	NA	NA	Bimac_3
PuBiY2	*Plesiopuntius*	*bimaculatus*	India: Yercaurd	11.783 N	78.21 E	87	OR667739	H31	NA	NA	NA	NA	NA	Bimac_3
PuMB1	*Puntius*	*mahecola*	India: Malvan, Bandivade	16.183 N	73.498 E	-	OR667740	-	OR666949	-	NA	-	NA	-
PuMB2	*Puntius*	*mahecola*	India: Malvan, Bandivade	16.183 N	73.498 E	-	OR667741	-	OR666950	-	NA	-	NA	-
PuUkn	*Puntius*	*mahecola*	India: Valankond	18.238 N	73.477 E	-	OR667742	-	NA	-	NA	-	NA	-
PuBiP1	*Puntius*	*mahecola*	India: Karnataka	14.498 N	74.865 E	-	OR667743	-	NA	-	NA	-	NA	-
PuBiS1	*Puntius*	*mahecola*	India: Shimoga	13.87 N	75.564 E	-	OR667744	-	NA	-	NA	-	NA	-
PuSob1	*Puntius*	*sophore*	India: West bengal	23.023 N	88.426 E	-	OR667745	-	NA	-	NA	-	NA	Sophore_2
PuSoCu2	*Puntius*	*sophore*	India: Cuddalore, Alappakkam	11.75 N	79.708 E	-	OR667746	-	NA	-	NA	-	NA	Sophore_2
PuSoKo1	*Puntius*	*sophore*	India: Karad	17.28 N	74.177 E	-	OR667747	-	NA	-	NA	-	NA	Sophore_2
PuSoKo2	*Puntius*	*sophore*	India: Karad	17.28 N	74.177 E	-	OR667748	-	NA	-	NA	-	NA	Sophore_2
PuTaK1	*Puntius*	*sophore*	India: Talakady, Karnataka	12.172 N	77.026 E	-	OR667749	-	NA	-	NA	-	NA	Sophore_2
PuTaK2	*Puntius*	*sophore*	India: Talakady, Karnataka	12.172 N	77.026 E	-	OR667750	-	NA	-	NA	-	NA	Sophore_2
PuTeg1	*Puntius*	*terio*	India: West Bengal	22.902 N	88.851 E	-	OR667751	-	NA	-	NA	-	NA	Sophore_4
PuTeg2	*Puntius*	*terio*	India: West Bengal	22.902 N	88.851 E	-	OR667752	-	NA	-	NA	-	NA	Sophore_4

Supplementary Table S3. Details of the sequences of Sri Lankan and Indian *Puntius* s.l. used in the haplotype network analyses with their localities, voucher references, and GenBank accession numbers.

Voucher	Genus	Species	Location	Latitude	Longitude	Locality	*cytb*	*cytb* haplotype	*cox1*	*cox1* haplotype	*rag1*	*rag1 haplotype*	*irbp*	Subclade	Source
DZ4355	*Puntius*	*thermalis*	Ranawarana wewa, Lunugamwehera: Kirindi Oya	6.4142 N	81.1308 E	13	OP880935	H2	OP855687	H3	OP881158	H1	NA	Sophore_6	Sudasinghe et al. (2023)
DZ4628	*Puntius*	*thermalis*	Bimpokunu ara, Rathabalagama, Hambegamuwa: Walawe	6.5267 N	80.9371 E	20	OP881030	H2	OP855585	H3	OP881150	H1	OP881066	Sophore_6	Sudasinghe et al. (2023)
DZ3027	*Puntius*	*thermalis*	Dellabada, Elapatha, Ratnapura: Kalu	6.6758 N	80.3537 E	22	OP880928	H3	OP855582	H2	NA	NA	NA	Sophore_6	Sudasinghe et al. (2023)
DZ3030	*Puntius*	*thermalis*	Dellabada, Elapatha, Ratnapura: Kalu	6.6758 N	80.3537 E	22	OP880929	H3	OP855581	H2	OP881149	H1	NA	Sophore_6	Sudasinghe et al. (2023)
DZ4610	*Puntius*	*thermalis*	Yudaganawa wewa, Buttala: Menik	6.7598 N	81.2255 E	27	OP880934	H2	OP855584	H3	OP881151	H1	OP881065	Sophore_6	Sudasinghe et al. (2023)
DZ4646	*Puntius*	*thermalis*	Alawala: Attanagalu	7.1201 N	80.1901 E	44	OP880927	H1	OP855583	H2	OP881157	H1	NA	Sophore_6	Sudasinghe et al. (2023)
DZ4580	*Puntius*	*thermalis*	Thummodara, Namal Oya, Gal Oya NP border: Gal Oya	7.2328 N	81.5372 E	50	OP880933	H1	OP855578	H1	OP881152	H1	OP881064	Sophore_6	Sudasinghe et al. (2023)
DZ1498	*Puntius*	*thermalis*	Ulhitiya Oya, Ulhitiya: Mahaweli	7.3799 N	81.0939 E	54	OP880931	H6	OP855579	H1	NA	NA	NA	Sophore_6	Sudasinghe et al. (2023)
DZ4860	*Puntius*	*thermalis*	Polonnaruwa: Mahaweli	7.9352 N	81.0112 E	60	OP880930	H1	OP855575	H1	OP881154	H1	OP881062	Sophore_6	Sudasinghe et al. (2023)
DZ4801	*Puntius*	*thermalis*	Maha Elagamuwa: Kala Oya	7.9898 N	80.6184 E	61	OP880924	H1	OP855573	H4	OP881156	H1	OP881060	Sophore_6	Sudasinghe et al. (2023)
DZ4870	*Puntius*	*thermalis*	Rajanganaya: Kala Oya	8.1415 N	80.2211 E	62	OP880925	H1	OP855576	H1	OP881153	H1	OP881063	Sophore_6	Sudasinghe et al. (2023)
DZ4871	*Puntius*	*thermalis*	Rajanganaya: Kala Oya	8.1415 N	80.2211 E	62	OP880926	H7	OP855577	H1	NA	NA	NA	Sophore_6	Sudasinghe et al. (2023)
DZ4148	*Puntius*	*thermalis*	Kantale: Mahaweli	8.2781 N	81.0697 E	64	OP880932	H5	OP855580	H1	OP881159	H1	NA	Sophore_6	Sudasinghe et al. (2023)
DZ4850	*Puntius*	*thermalis*	Nelum Wewa, Eluwankulama: Kala Oya	8.2798 N	79.8754 E	65	OP880923	H4	OP855574	H2	OP881155	H1	OP881061	Sophore_6	Sudasinghe et al. (2023)
DZ3172	*Puntius*	*thermalis*	Polonnaruwa, Mahaweli basin	7.8621 N	80.9558 E	75	MT732736	H6	MT738920	H1	MT732771	H1	NA	Sophore_6	Sudasinghe et al. (2020)
RZM-FISH-267	*Puntius*	*thermalis*	Thalagaha	6.07 N	80.24 E	68	NA	NA	KT719181	H3	NA	NA	NA	Sophore_6	GenBank
RZM-FISH-323	*Puntius*	*thermalis*	Godapitiya	6.12 N	80.48 E	69	NA	NA	KT719183	H3	NA	NA	NA	Sophore_6	GenBank
RZM-FISH-324	*Puntius*	*thermalis*	Godapitiya	6.12 N	80.48 E	69	NA	NA	KT719184	H3	NA	NA	NA	Sophore_6	GenBank
RZM 221	*Puntius*	*thermalis*	Deduru Oya	7.62 N	79.89 E	74	NA	NA	MG797668	H3	NA	NA	NA	Sophore_6	GenBank
RZM-FISH-405	*Puntius*	*thermalis*	Anuradhapura	8.34 N	80.39 E	77	NA	NA	KT719185	H1	NA	NA	NA	Sophore_6	GenBank
PuSuCu1	*Puntius*	*thermalis*	India: Cuddalore, Alappakkam	11.75 N	79.708 E	88	Done	H8	NA	NA	NA	NA	NA	Sophore_6	This study
DZ1456	*Puntius*	*dorsalis*	Pambahinna, Belihuloya: Walawe	6.6969 N	80.7761 E	24	OP880961	H1	OP855596	H1	OP881139	H1	NA	Dorsalis_1	Sudasinghe et al. (2023)
DZ4598	*Puntius*	*dorsalis*	Meegahayaya, Badalkumbura: Menik	6.8709 N	81.2328 E	30	OP880959	H2	OP855598	H2	OP881138	H2	NA	Dorsalis_1	Sudasinghe et al. (2023)
DZ4599	*Puntius*	*dorsalis*	Meegahayaya, Badalkumbura: Menik	6.8709 N	81.2328 E	30	OP880960	H2	OP855597	H2	NA	NA	NA	Dorsalis_1	Sudasinghe et al. (2023)
DZ3217	*Puntius*	*dorsalis*	Miyanawita, Deraniyagala: Kelani	6.8949 N	80.3549 E	31	OP880942	H8	OP855592	H8	OP881142	H3	NA	Dorsalis_2	Sudasinghe et al. (2023)
DZ3218	*Puntius*	*dorsalis*	Miyanawita, Deraniyagala: Kelani	6.8949 N	80.3549 E	31	OP880943	H9	OP855591	H8	OP881141	H4	NA	Dorsalis_2	Sudasinghe et al. (2023)
DZ4993	*Puntius*	*dorsalis*	near Yogama pool: Kelani	6.9245 N	80.2746 E	34	OP880940	H9	OP855593	H7	OP881144	H3	NA	Dorsalis_2	Sudasinghe et al. (2023)
DZ4994	*Puntius*	*dorsalis*	near Yogama pool: Kelani	6.9245 N	80.2746 E	34	OP880941	H7	OP855594	H8	OP881143	H3	NA	Dorsalis_2	Sudasinghe et al. (2023)
DZ4641	*Puntius*	*dorsalis*	Alawala: Attanagalu	7.1201 N	80.1901 E	44	OP880938	H6	OP855589	H6	OP881147	H4	NA	Dorsalis_2	Sudasinghe et al. (2023)
DZ4642	*Puntius*	*dorsalis*	Alawala: Attanagalu	7.1201 N	80.1901 E	44	OP880939	H9	OP855590	H8	NA	NA	NA	Dorsalis_2	Sudasinghe et al. (2023)
DZ3145	*Puntius*	*dorsalis*	Pannalthota, Uggoda, Aranayake: Maa Oya	7.1428 N	80.4686 E	46	OP881029	H5	OP855586	H5	OP881148	H4	NA	Dorsalis_2	Sudasinghe et al. (2023)
DZ4867	*Puntius*	*dorsalis*	Rajanganaya: Kala Oya	8.1415 N	80.2211 E	62	OP880936	H4	OP855587	H3	OP881146	H3	NA	Dorsalis_2	Sudasinghe et al. (2023)
DZ4868	*Puntius*	*dorsalis*	Rajanganaya: Kala Oya	8.1415 N	80.2211 E	62	OP880937	H3	OP855588	H4	OP881145	H3	NA	Dorsalis_2	Sudasinghe et al. (2023)
DZ3536	*Puntius*	*dorsalis*	Kalu ganga, Laggala-Pallegama bridge: Mahaweli	7.5456 N	80.8225 E	58	NA	NA	OP855595	H6	OP881140	H3	NA	Dorsalis_2	Sudasinghe et al. (2023)
DZ4353	*Puntius*	*dorsalis*	Ranawarana wewa, Lunugamwehera: Kirindi Oya	6.4142 N	81.1308 E	13	OP880958	H10	OP855612	H9	OP881137	H5	NA	Dorsalis_3	Sudasinghe et al. (2023)
DZ4354	*Puntius*	*dorsalis*	Ranawarana wewa, Lunugamwehera: Kirindi Oya	6.4142 N	81.1308 E	13	OP880957	H10	OP855611	H9	NA	NA	NA	Dorsalis_3	Sudasinghe et al. (2023)
DZ4626	*Puntius*	*dorsalis*	Bimpokunu ara, Rathabalagama, Hambegamuwa: Walawe	6.5267 N	80.9371 E	20	OP880953	H14	OP855602	H13	OP881132	H5	NA	Dorsalis_3	Sudasinghe et al. (2023)
DZ4627	*Puntius*	*dorsalis*	Bimpokunu ara, Rathabalagama, Hambegamuwa: Walawe	6.5267 N	80.9371 E	20	OP880952	H15	OP855603	H13	NA	NA	NA	Dorsalis_3	Sudasinghe et al. (2023)
DZ4607	*Puntius*	*dorsalis*	Yudaganawa wewa, Buttala: Menik	6.7598 N	81.2255 E	27	OP880956	H10	OP855605	H9	OP881133	H5	NA	Dorsalis_3	Sudasinghe et al. (2023)
DZ4590	*Puntius*	*dorsalis*	Bellan Oya, Nakkala-Passara road: Kumbukkan	6.9458 N	81.2874 E	35	OP880955	H11	OP855610	H9	OP881134	H6	NA	Dorsalis_3	Sudasinghe et al. (2023)
DZ4591	*Puntius*	*dorsalis*	Bellan Oya, Nakkala-Passara road: Kumbukkan	6.9458 N	81.2874 E	35	OP880954	H11	OP855609	H9	NA	NA	NA	Dorsalis_3	Sudasinghe et al. (2023)
DZ4549	*Puntius*	*dorsalis*	Diyakobala road, Kotagama: Gal Oya	7.132 N	81.1818 E	45	OP880951	H13	OP855608	H12	OP881136	H5	NA	Dorsalis_3	Sudasinghe et al. (2023)
DZ4550	*Puntius*	*dorsalis*	Diyakobala road, Kotagama: Gal Oya	7.132 N	81.1818 E	45	OP880950	H13	OP855607	H12	NA	NA	NA	Dorsalis_3	Sudasinghe et al. (2023)
DZ4573	*Puntius*	*dorsalis*	Gal wala, spill of Senanatake samudra, Iginiyagala: Gal Oya	7.2277 N	81.5344 E	49	NA	NA	OP855606	H12	OP881135	H5	NA	Dorsalis_3	Sudasinghe et al. (2023)
DZ3085	*Puntius*	*dorsalis*	Mahaweli River at Getambe, Kandy: Mahaweli	7.271 N	80.6038 E	52	OP880946	H17	OP855600	H13	NA	NA	NA	Dorsalis_3	Sudasinghe et al. (2023)
DZ3435	*Puntius*	*dorsalis*	Owala mini hydro plant, Sudu ganga, Naula: Mahaweli	7.5178 N	80.6431 E	56	OP880948	H17	OP855599	H13	NA	NA	NA	Dorsalis_3	Sudasinghe et al. (2023)
DZ4003	*Puntius*	*dorsalis*	Magalavatavan Aru, Nuwaragalathenna: Magalavatavan Aru	7.5179 N	81.5002 E	57	NA	NA	OP855604	H13	NA	NA	NA	Dorsalis_3	Sudasinghe et al. (2023)
DZ3184	*Puntius*	*dorsalis*	below Dora dahaya sorowwa, Polonnaruwa: Mahaweli	7.8715 N	80.9678 E	59	OP880947	H17	OP855601	H13	OP881129	H5	NA	Dorsalis_3	Sudasinghe et al. (2023)
DZ5113	*Puntius*	*dorsalis*	Thulawelliya, Kanadara Oya: Malwathu Oya	8.5091 N	80.4229 E	66	OP880945	H12	OP855613	H10	OP881131	H5	OP881046	Dorsalis_3	Sudasinghe et al. (2023)
DZ5114	*Puntius*	*dorsalis*	Thulawelliya, Kanadara Oya: Malwathu Oya	8.5091 N	80.4229 E	66	OP880944	H12	OP855686	H10	OP881130	H5	OP881047	Dorsalis_3	Sudasinghe et al. (2023)
DZ4858	*Puntius*	*dorsalis*	Polonnaruwa: Mahaweli	7.9352 N	81.0112 E	60	OP880949	H16	NA	NA	OP881128	H5	OP881045	Dorsalis_3	Sudasinghe et al. (2023)
DZ3152	*Puntius*	*dorsalis*	Polonnaruwa, Mahaweli basin	7.8621 N	80.9558 E	75	MT732735	H15	MT738919	H14	MT732770	H5	NA	Dorsalis_3	Sudasinghe et al. (2020)
DZ4487	*Puntius*	*kamalika*	Ugudu dola, Opatha: Nilwala	6.2669 N	80.4023 E	6	OP880962	H4	OP855685	H1	OP881098	H1	NA	-	Sudasinghe et al. (2023)
DZ4488	*Puntius*	*kamalika*	Ugudu dola, Opatha: Nilwala	6.2669 N	80.4023 E	6	OP880963	H4	OP855619	H1	OP881099	H1	NA	-	Sudasinghe et al. (2023)
DZ3206	*Puntius*	*kamalika*	Mahakanadarawa ela: Bentara	6.4372 N	80.1282 E	14	OP880964	H4	OP855616	H1	OP881095	H1	NA	-	Sudasinghe et al. (2023)
DZ3053	*Puntius*	*kamalika*	Mawathgama, Homagama: Bolgoda	6.8174 N	80.0001 E	29	OP880966	H3	OP855618	H1	OP881097	H1	NA	-	Sudasinghe et al. (2023)
DZ3054	*Puntius*	*kamalika*	Mawathgama, Homagama: Bolgoda	6.8174 N	80.0001 E	29	OP880965	H4	OP855617	H1	OP881096	H1	NA	-	Sudasinghe et al. (2023)
DZ5117	*Puntius*	*kamalika*	Thulawelliya, Kanadara Oya: Malwathu Oya	8.5091 N	80.4229 E	66	OP880967	H2	OP855614	H1	OP881100	H1	NA	-	Sudasinghe et al. (2023)
DZ5118	*Puntius*	*kamalika*	Thulawelliya, Kanadara Oya: Malwathu Oya	8.5091 N	80.4229 E	66	OP881028	H1	OP855615	H1	NA	NA	NA	-	Sudasinghe et al. (2023)
DZ4473	*Puntius*	*kelumi*	Ugudu dola, Opatha: Nilwala	6.2669 N	80.4023 E	6	OP880975	H1	OP855630	H3	OP881126	H3	NA	Kelumi_1	Sudasinghe et al. (2023)
DZ5053	*Puntius*	*kelumi*	Kotapola dola, under Kotapola bridge: Nilwala	6.2949 N	80.544 E	7	OP880976	H1	OP855628	H2	OP881122	H3	NA	Kelumi_1	Sudasinghe et al. (2023)
DZ5054	*Puntius*	*kelumi*	Kotapola dola, under Kotapola bridge: Nilwala	6.2949 N	80.544 E	7	OP881027	H1	OP855629	H1	OP881121	H3	NA	Kelumi_1	Sudasinghe et al. (2023)
DZ4980	*Puntius*	*kelumi*	near Hiniduma Rest House, Hiniduma: Gin	6.3033 N	80.3193 E	8	OP880974	H3	OP855626	H4	NA	NA	NA	Kelumi_1	Sudasinghe et al. (2023)
DZ4981	*Puntius*	*kelumi*	near Hiniduma Rest House, Hiniduma: Gin	6.3033 N	80.3193 E	8	OP880973	H4	OP855627	H4	OP881123	H3	NA	Kelumi_1	Sudasinghe et al. (2023)
DZ4938	*Puntius*	*kelumi*	Kottawa Forest Reserve, Galle: Gin	6.0986 N	80.3146 E	2	OP880972	H7	OP855625	H5	OP881124	H1	NA	Kelumi_2	Sudasinghe et al. (2023)
DZ3098	*Puntius*	*kelumi*	Thundola, Horawala: Bentara	6.475 N	80.1524 E	16	NA	NA	OP855624	H6	NA	NA	NA	Kelumi_2	Sudasinghe et al. (2023)
DZ3221	*Puntius*	*kelumi*	Miyanawita, Deraniyagala: Kelani	6.8949 N	80.3549 E	31	OP880968	H5	OP855684	H9	OP881118	H2	NA	Kelumi_2	Sudasinghe et al. (2023)

DZ5154	*Puntius*	*kelumi*	Lenagala: Attanagalu	7.1047 N	80.2177 E	43	OP880969	H6	OP855631	H8	OP881120	H2	NA	Kelumi_2	Sudasinghe et al. (2023)
DZ5155	*Puntius*	*kelumi*	Lenagala: Attanagalu	7.1047 N	80.2177 E	43	OP880970	H6	OP855632	H8	OP881119	H2	NA	Kelumi_2	Sudasinghe et al. (2023)
DZ5156	*Puntius*	*kelumi*	Lenagala: Attanagalu	7.1047 N	80.2177 E	43	OP880971	H6	OP855633	H7	NA	NA	NA	Kelumi_2	Sudasinghe et al. (2023)
DZ4771	*Puntius*	*kelumi*	Runakanda Forest Reserve, Mathugama: Kalu	6.4548 N	80.3263 E	15	NA	NA	OP855620	H11	OP881125	H2	NA	Kelumi_3	Sudasinghe et al. (2023)
DZ4728	*Puntius*	*kelumi*	Indikada dola, Weralugahamula, Rakwana: Kalu	6.4876 N	80.5993 E	17	NA	NA	OP855622	H12	OP881127	H2	NA	Kelumi_3	Sudasinghe et al. (2023)
DZ4729	*Puntius*	*kelumi*	Indikada dola, Weralugahamula, Rakwana: Kalu	6.4876 N	80.5993 E	17	NA	NA	OP855623	H12	NA	NA	NA	Kelumi_3	Sudasinghe et al. (2023)
DZ3361	*Puntius*	*kelumi*	Athwelthota: Kalu	6.5382 N	80.2901 E	21	NA	NA	OP855621	H10	NA	NA	NA	Kelumi_3	Sudasinghe et al. (2023)
DZ1359	*Rohanella* gen. nov.	*titteya*	Hiyare Forest, Galle: Gin	6.0567 N	80.3195 E	1	OP881008	H15	OP855666	H14	NA	NA	NA	-	Sudasinghe et al. (2023)
DZ4913	*Rohanella*	*titteya*	Kottawa Forest Reserve, Galle: Gin	6.0986 N	80.3146 E	2	OP881012	H14	OP855668	H17	OP881104	H5	OP881040	-	Sudasinghe et al. (2023)
DZ4914	*Rohanella*	*titteya*	Kottawa Forest Reserve, Galle: Gin	6.0986 N	80.3146 E	2	OP881010	H16	OP855667	H15	OP881103	H5	OP881041	-	Sudasinghe et al. (2023)
DZ4915	*Rohanella*	*titteya*	Kottawa Forest Reserve, Galle: Gin	6.0986 N	80.3146 E	2	OP881011	H14	OP855669	H17	NA	NA	NA	-	Sudasinghe et al. (2023)
DZ4916	*Rohanella*	*titteya*	Kottawa Forest Reserve, Galle: Gin	6.0986 N	80.3146 E	2	OP881009	H13	OP855670	H16	NA	NA	NA	-	Sudasinghe et al. (2023)
DZ4905	*Rohanella*	*titteya*	stream near Welihena Forest Reserve: Nilwala	6.1101 N	80.5131 E	3	OP881015	H18	OP855671	H18	OP881106	H5	OP881038	-	Sudasinghe et al. (2023)
DZ4906	*Rohanella*	*titteya*	stream near Welihena Forest Reserve: Nilwala	6.1101 N	80.5131 E	3	OP881014	H19	OP855672	H18	OP881105	H2	OP881039	-	Sudasinghe et al. (2023)
DZ4907	*Rohanella*	*titteya*	stream near Welihena Forest Reserve: Nilwala	6.1101 N	80.5131 E	3	OP881013	H18	OP855673	H18	NA	NA	NA	-	Sudasinghe et al. (2023)
DZ4461	*Rohanella*	*titteya*	Dediyagala, Akuressa: Nilwala	6.1741 N	80.4195 E	4	OP881026	H17	OP855683	H21	OP881114	H2	NA	-	Sudasinghe et al. (2023)
DZ4982	*Rohanella*	*titteya*	Kaludola, Pitigala: Bentara	6.3749 N	80.2396 E	12	OP881007	H11	OP855664	H13	OP881102	H6	OP881042	-	Sudasinghe et al. (2023)
DZ4983	*Rohanella*	*titteya*	Kaludola, Pitigala: Bentara	6.3749 N	80.2396 E	12	OP881006	H12	OP855665	H12	OP881101	H6	OP881043	-	Sudasinghe et al. (2023)
DZ4765	*Rohanella*	*titteya*	Runakanda Forest Reserve, Mathugama: Kalu	6.4548 N	80.3263 E	15	OP881016	H5	OP855661	H6	NA	NA	NA	-	Sudasinghe et al. (2023)
DZ3108	*Rohanella*	*titteya*	Thundola, Horawala: Bentara	6.475 N	80.1524 E	16	OP881005	H10	OP855663	H11	OP881115	H5	NA	-	Sudasinghe et al. (2023)
DZ3046	*Rohanella*	*titteya*	Dellabada, Elapatha, Ratnapura: Kalu	6.6789 N	80.351 E	23	OP881000	H4	OP855662	H5	NA	NA	NA	-	Sudasinghe et al. (2023)
DZ1338	*Rohanella*	*titteya*	Dombagaskanda Forest Reserve, Ingiriya: Kalu	6.7245 N	80.1606 E	26	OP881002	H3	OP855655	H3	NA	NA	NA	-	Sudasinghe et al. (2023)
DZ1339	*Rohanella*	*titteya*	Dombagaskanda Forest Reserve, Ingiriya: Kalu	6.7245 N	80.1606 E	26	OP880997	H6	OP855657	H5	OP881110	H2	OP881034	-	Sudasinghe et al. (2023)
DZ1340	*Rohanella*	*titteya*	Dombagaskanda Forest Reserve, Ingiriya: Kalu	6.7245 N	80.1606 E	26	OP881001	H3	OP855656	H3	OP881109	H2	OP881035	-	Sudasinghe et al. (2023)
DZ1341	*Rohanella*	*titteya*	Dombagaskanda Forest Reserve, Ingiriya: Kalu	6.7245 N	80.1606 E	26	OP881003	H1	OP855660	H1	NA	NA	NA	-	Sudasinghe et al. (2023)
DZ1342	*Rohanella*	*titteya*	Dombagaskanda Forest Reserve, Ingiriya: Kalu	6.7245 N	80.1606 E	26	OP880998	H6	OP855658	H5	NA	NA	NA	-	Sudasinghe et al. (2023)
DZ1348	*Rohanella*	*titteya*	Dombagaskanda Forest Reserve, Ingiriya: Kalu	6.7245 N	80.1606 E	26	OP880999	H6	OP855659	H5	NA	NA	NA	-	Sudasinghe et al. (2023)
DZ3397	*Rohanella*	*titteya*	stream inside Induruwa forest, Gilimale: Kalu	6.7641 N	80.4293 E	28	OP881004	H2	OP855654	H2	OP881116	H1	OP881044	-	Sudasinghe et al. (2023)
DZ1343	*Rohanella*	*titteya*	Welikenna: Kelani	6.8969 N	80.1518 E	32	OP880993	H8	OP855649	H9	NA	NA	NA	-	Sudasinghe et al. (2023)
DZ1344	*Rohanella*	*titteya*	Welikenna: Kelani	6.8969 N	80.1518 E	32	OP880992	H8	OP855650	H9	NA	NA	NA	-	Sudasinghe et al. (2023)
DZ1345	*Rohanella*	*titteya*	Welikenna: Kelani	6.8969 N	80.1518 E	32	OP880994	H8	OP855648	H9	NA	NA	NA	-	Sudasinghe et al. (2023)
DZ1346	*Rohanella*	*titteya*	Welikenna: Kelani	6.8969 N	80.1518 E	32	OP880991	H8	OP855651	H9	OP881108	H3	OP881036	-	Sudasinghe et al. (2023)
DZ1347	*Rohanella*	*titteya*	Welikenna: Kelani	6.8969 N	80.1518 E	32	OP880990	H8	OP855652	H9	OP881107	H3	OP881037	-	Sudasinghe et al. (2023)
DZ3052	*Rohanella*	*titteya*	Magammana, Dehiowita: Kelani	7.0072 N	80.2635 E	36	OP880995	H7	OP855653	H8	NA	NA	NA	-	Sudasinghe et al. (2023)
DZ3227	*Rohanella*	*titteya*	Sea-Forth, Balantota: Mahaweli	7.0333 N	80.475 E	37	OP880988	H22	OP855639	H19	NA	NA	NA	-	Sudasinghe et al. (2023)
DZ3228	*Rohanella*	*titteya*	Sea-Forth, Balantota: Mahaweli	7.0333 N	80.475 E	37	OP880977	H21	OP855634	H20	NA	NA	NA	-	Sudasinghe et al. (2023)
DZ3229	*Rohanella*	*titteya*	Sea-Forth, Balantota: Mahaweli	7.0333 N	80.475 E	37	OP880978	H21	OP855635	H20	OP881112	H5	OP881032	-	Sudasinghe et al. (2023)
DZ3230	*Rohanella*	*titteya*	Sea-Forth, Balantota: Mahaweli	7.0333 N	80.475 E	37	OP880987	H22	OP855640	H19	OP881111	H5	OP881033	-	Sudasinghe et al. (2023)
DZ3231	*Rohanella*	*titteya*	Sea-Forth, Balantota: Mahaweli	7.0333 N	80.475 E	37	OP880982	H20	OP855646	H18	NA	NA	NA	-	Sudasinghe et al. (2023)
DZ3232	*Rohanella*	*titteya*	Sea-Forth, Balantota: Mahaweli	7.0333 N	80.475 E	37	OP880996	H6	OP855641	H19	NA	NA	NA	-	Sudasinghe et al. (2023)
DZ3247	*Rohanella*	*titteya*	Sea-Forth, Balantota: Mahaweli	7.0333 N	80.475 E	37	OP880979	H21	OP855636	H20	NA	NA	NA	-	Sudasinghe et al. (2023)
DZ3248	*Rohanella*	*titteya*	Sea-Forth, Balantota: Mahaweli	7.0333 N	80.475 E	37	OP880986	H22	OP855642	H19	NA	NA	NA	-	Sudasinghe et al. (2023)
DZ3250	*Rohanella*	*titteya*	Sea-Forth, Balantota: Mahaweli	7.0333 N	80.475 E	37	OP880985	H22	OP855643	H19	NA	NA	NA	-	Sudasinghe et al. (2023)
DZ3251	*Rohanella*	*titteya*	Sea-Forth, Balantota: Mahaweli	7.0333 N	80.475 E	37	OP880981	H21	OP855637	H20	NA	NA	NA	-	Sudasinghe et al. (2023)
DZ3252	*Rohanella*	*titteya*	Sea-Forth, Balantota: Mahaweli	7.0333 N	80.475 E	37	OP880983	H22	OP855644	H19	OP881117	H5	NA	-	Sudasinghe et al. (2023)
DZ3253	*Rohanella*	*titteya*	Sea-Forth, Balantota: Mahaweli	7.0333 N	80.475 E	37	OP880980	H21	OP855638	H20	NA	NA	NA	-	Sudasinghe et al. (2023)
DZ3254	*Rohanella*	*titteya*	Sea-Forth, Balantota: Mahaweli	7.0333 N	80.475 E	37	OP880984	H22	OP855645	H19	NA	NA	NA	-	Sudasinghe et al. (2023)
DZ4280	*Rohanella*	*titteya*	Kandumulla, Yakkala: Attanagalu Oya	7.0792 N	80.0732 E	42	OP880989	H9	OP855647	H10	OP881113	H4	NA	-	Sudasinghe et al. (2023)
DZ3224	*Rohanella*	*titteya*	Sri Lanka: Deraniyagala, Kelani basin	6.8948 N	80.3549 E	71	MT732737	H6	MT738921	H7	MT732772	H2	NA	-	Sudasinghe et al. (2020)
DZ3347	*Bhava* gen. nov.	*vittata*	Wannigoda, Kukulegama south, Kalawana: Kalu	6.5114 N	80.37 E	19	OP881025	H1	OP855681	H1	NA	NA	NA	-	Sudasinghe et al. (2023)
DZ3028	*Bhava*	*vittata*	Dellabada, Elapatha, Ratnapura: Kalu	6.6758 N	80.3537 E	22	OP881024	H1	OP855682	H1	NA	NA	NA	-	Sudasinghe et al. (2023)
DZ3060	*Bhava*	*vittata*	Mawathgama, Homagama: Bolgoda	6.8174 N	80.0001 E	29	OP881023	H5	OP855680	H3	NA	NA	NA	-	Sudasinghe et al. (2023)
DZ5139	*Bhava*	*vittata*	Kandumulla: Attanagalu Oya	7.0687 N	80.0701 E	41	OP881022	H6	OP855679	H3	OP881161	H2	NA	-	Sudasinghe et al. (2023)
DZ5175	*Bhava*	*vittata*	Nelum Wewa, Eluwankulama: Kala Oya	8.2798 N	79.8754 E	65	OP881018	H3	OP855674	H2	OP881164	H2	NA	-	Sudasinghe et al. (2023)
DZ5106	*Bhava*	*vittata*	Thulawelliya, Kanadara Oya: Malwathu Oya	8.5091 N	80.4229 E	66	OP881017	H3	OP855678	H2	OP881160	H2	NA	-	Sudasinghe et al. (2023)
DZ5160	*Bhava*	*vittata*	Pooneryn: Mandekal Aru	9.5032 N	80.2124 E	67	OP881021	H4	OP855675	H2	OP881162	H2	NA	-	Sudasinghe et al. (2023)
DZ5162	*Bhava*	*vittata*	Pooneryn: Mandekal Aru	9.5032 N	80.2124 E	67	OP881020	H4	OP855676	H2	OP881163	H2	NA	-	Sudasinghe et al. (2023)
DZ5163	*Bhava*	*vittata*	Pooneryn: Mandekal Aru	9.5032 N	80.2124 E	67	OP881019	H4	OP855677	H2	NA	NA	NA	-	Sudasinghe et al. (2023)
DZ3290	*Bhava*	*vittata*	Remuna, Kalu basin	6.6857 N	80.0649 E	70	MT732740	H2	MT738924	H4	MT732775	H1	NA	-	Sudasinghe et al. (2020)
DZ3180	*Bhava*	*vittata*	Polonnaruwa, Mahaweli basin	7.8703 N	80.9569 E	76	MT732739	H7	MT738923	H2	MT732774	H2	NA	-	Sudasinghe et al. (2020)
M-CCI-PV1	*Bhava*	*vittata*	India	10.21 N	76.15 E	78	NA	NA	HE801572	H6	NA	NA	NA	-	GenBank
NBFGR:PVT1	*Bhava*	*vittata*	India	10.3 N	76.33 E	79	JQ795454	H8	JX181844	H8	NA	NA	NA	-	GenBank
NBFGR:PVT4	*Bhava*	*vittata*	India	10.3 N	76.33 E	79	JQ795457	H9	JX181845	H7	NA	NA	NA	-	GenBank
NBFGR:PVT2	*Bhava*	*vittata*	India	10.3 N	76.33 E	79	JQ795455	H8	JX181846	H7	NA	NA	NA	-	GenBank
NBFGR:PVT3	*Bhava*	*vittata*	India	10.3 N	76.33 E	79	JQ795456	H9	JX181847	H8	NA	NA	NA	-	GenBank
NBFGR:PVT5	*Bhava*	*vittata*	India	10.3 N	76.33 E	79	JQ795458	H10	JX181848	H9	NA	NA	NA	-	GenBank
ZSI-WRC P/4803	*Bhava*	*vittata*	India: Kabani River, Madekere	12.095 N	76.375 E	80	NA	NA	KX073603	H5	NA	NA	NA	-	GenBank
DZ3319	*Plesiopuntius* gen. nov.	*bimaculatus*	Sapugaha dola, Rammale: Urubokka Oya	6.2416 N	80.6455 E	5	OP880922	H3	OP855688	H3	OP881071	H1	NA	Bimac_1	Sudasinghe et al. (2023)

DZ3320	*Plesiopuntius*	*bimaculatus*	Sapugaha dola, Rammale: Urubokka Oya	6.2416 N	80.6455 E	5	OP881031	H3	OP855572	H3	OP881072	H1	NA	Bimac_1	Sudasinghe et al. (2023)
DZ3395	*Plesiopuntius*	*bimaculatus*	stream inside Induruwa forest, Gilimale: Kalu	6.7641 N	80.4293 E	28	OP880920	H1	OP855571	H2	NA	NA	NA	Bimac_1	Sudasinghe et al. (2023)
DZ3396	*Plesiopuntius*	*bimaculatus*	stream inside Induruwa forest, Gilimale: Kalu	6.7641 N	80.4293 E	28	OP880921	H2	OP855570	H1	OP881070	H1	NA	Bimac_1	Sudasinghe et al. (2023)
DZ5055	*Plesiopuntius*	*bimaculatus*	Beralapanathara: Nilwala	6.3221 N	80.5972 E	9	OP880916	H4	OP855569	H4	OP881075	H6	OP881051	Bimac_2	Sudasinghe et al. (2023)
DZ3323	*Plesiopuntius*	*bimaculatus*	Pallegama, Deniyaya: Gin	6.3416 N	80.5397 E	10	OP880918	H7	OP855568	H4	OP881073	H6	NA	Bimac_2	Sudasinghe et al. (2023)
DZ3330	*Plesiopuntius*	*bimaculatus*	stream near SDM Guest Houses, Deniyaya: Gin	6.3449 N	80.5619 E	11	OP880919	H7	OP855567	H4	OP881074	H6	NA	Bimac_2	Sudasinghe et al. (2023)
DZ3345	*Plesiopuntius*	*bimaculatus*	Weralugahamula, Rakwana: Kalu	6.4887 N	80.5991 E	18	OP880915	H6	OP855566	H4	NA	NA	NA	Bimac_2	Sudasinghe et al. (2023)
DZ1455	*Plesiopuntius*	*bimaculatus*	Mutthethugamuwa bridge, Pambahinna, Belihul Oya: Walawe	6.7018 N	80.8006 E	25	OP880916	H5	OP855565	H4	OP881084	H6	OP881057	Bimac_2	Sudasinghe et al. (2023)
DZ4606	*Plesiopuntius*	*bimaculatus*	Yudaganawa wewa, Buttala: Menik	6.7598 N	81.2255 E	27	OP880909	H11	OP855559	H7	OP881085	H6	NA	Bimac_2	Sudasinghe et al. (2023)
DZ4235	*Plesiopuntius*	*bimaculatus*	Kanaweralla, towards Bibilegama, Passara: Menik	6.9059 N	81.1343 E	33	OP880910	H11	OP855560	H7	OP881086	H6	NA	Bimac_2	Sudasinghe et al. (2023)
DZ4588	*Plesiopuntius*	*bimaculatus*	Bellan Oya, Nakkala-Passara road: Kumbukkan	6.9458 N	81.2874 E	35	OP880911	H10	OP855562	H6	NA	NA	NA	Bimac_2	Sudasinghe et al. (2023)
DZ4220	*Plesiopuntius*	*bimaculatus*	Kiri Oya, Uda kiruwa: Kumbukkan	7.0526 N	81.2321 E	38	OP880914	H9	OP855564	H5	OP881090	H6	NA	Bimac_2	Sudasinghe et al. (2023)
DZ4221	*Plesiopuntius*	*bimaculatus*	Kiri Oya, Uda kiruwa: Kumbukkan	7.0526 N	81.2321 E	38	OP880913	H9	OP855563	H5	NA	NA	NA	Bimac_2	Sudasinghe et al. (2023)
DZ4215	*Plesiopuntius*	*bimaculatus*	28th post, Ibbanna Oya, Lunugala: Gal Oya	7.0609 N	81.2052 E	40	OP880912	H8	OP855561	H6	OP881091	H6	NA	Bimac_2	Sudasinghe et al. (2023)
DZ4559	*Plesiopuntius*	*bimaculatus*	Diyakobala road, Kotagama: Gal Oya	7.132 N	81.1818 E	45	OP880907	H12	OP855557	H9	OP881087	H6	NA	Bimac_2	Sudasinghe et al. (2023)
DZ4206	*Plesiopuntius*	*bimaculatus*	Hepola Oya, Kuragammala, Bibile: Mahaweli	7.1741 N	81.1534 E	47	OP880904	H12	OP855554	H9	NA	NA	NA	Bimac_2	Sudasinghe et al. (2023)
DZ4207	*Plesiopuntius*	*bimaculatus*	Hepola Oya, Kuragammala, Bibile: Mahaweli	7.1741 N	81.1534 E	47	OP880903	H13	OP855553	H9	NA	NA	NA	Bimac_2	Sudasinghe et al. (2023)
DZ3697	*Plesiopuntius*	*bimaculatus*	Mahadorowwa campsite, Nilgala, Gal Oya NP: Gal oya	7.1818 N	81.4063 E	48	OP880906	H12	OP855556	H9	OP881088	H5	OP881055	Bimac_2	Sudasinghe et al. (2023)
DZ3698	*Plesiopuntius*	*bimaculatus*	Mahadorowwa campsite, Nilgala, Gal Oya NP: Gal oya	7.1818 N	81.4063 E	48	OP880905	H12	OP855555	H9	OP881089	H6	OP881056	Bimac_2	Sudasinghe et al. (2023)
DZ4002	*Plesiopuntius*	*bimaculatus*	Magalavatavan Aru, Nuwaragalathenna: Magalavatavan Aru	7.5179 N	81.5002 E	57	OP880908	H14	OP855558	H8	OP881083	H6	NA	Bimac_2	Sudasinghe et al. (2023)
DZ3050	*Plesiopuntius*	*bimaculatus*	Nakadawala ela, Humbaswalana, Ruwanwella: Kelani	7.056 N	80.2363 E	39	OP880897	H26	OP855547	H17	OP881078	H6	OP881058	Bimac_3	Sudasinghe et al. (2023)
DZ5142	*Plesiopuntius*	*bimaculatus*	Kandumulla: Attanagalu Oya	7.0687 N	80.0701 E	41	OP880895	H25	OP855546	H16	OP881077	H2	OP881053	Bimac_3	Sudasinghe et al. (2023)
DZ5150	*Plesiopuntius*	*bimaculatus*	Lenagala: Attanagalu	7.1047 N	80.2177 E	43	OP880896	H23	OP855545	H16	OP881076	H6	OP881052	Bimac_3	Sudasinghe et al. (2023)
DZ4532	*Plesiopuntius*	*bimaculatus*	Molagoda, Kegalle: Maa Oya	7.2563 N	80.3913 E	51	OP880894	H24	OP855543	H16	OP881079	H6	NA	Bimac_3	Sudasinghe et al. (2023)
DZ4533	*Plesiopuntius*	*bimaculatus*	Molagoda, Kegalle: Maa Oya	7.2563 N	80.3913 E	51	OP880893	H23	OP855544	H16	NA	NA	NA	Bimac_3	Sudasinghe et al. (2023)
DZ4812	*Plesiopuntius*	*bimaculatus*	Mahawala, Uda dumbara: Mahaweli	7.3043 N	80.942 E	53	OP880899	H20	OP855549	H17	OP881094	H5	OP881049	Bimac_3	Sudasinghe et al. (2023)
DZ4813	*Plesiopuntius*	*bimaculatus*	Mahawala, Uda dumbara: Mahaweli	7.3043 N	80.942 E	53	OP880900	H21	OP855550	H14	NA	NA	NA	Bimac_3	Sudasinghe et al. (2023)
DZ1499	*Plesiopuntius*	*bimaculatus*	Ulhitiya Oya, Ulhitiya: Mahaweli	7.3799 N	81.0939 E	54	OP880901	H19	OP855552	H17	NA	NA	NA	Bimac_3	Sudasinghe et al. (2023)
DZ4502	*Plesiopuntius*	*bimaculatus*	Weuda: Deduru	7.4132 N	80.4982 E	55	OP880891	H23	OP855542	H16	OP881080	H6	NA	Bimac_3	Sudasinghe et al. (2023)
DZ4503	*Plesiopuntius*	*bimaculatus*	Weuda: Deduru	7.4132 N	80.4982 E	55	OP880892	H23	OP855541	H16	NA	NA	NA	Bimac_3	Sudasinghe et al. (2023)
DZ3179	*Plesiopuntius*	*bimaculatus*	below Dora dahaya sorowwa, Polonnaruwa: Mahaweli	7.8715 N	80.9678 E	59	OP880902	H22	OP855551	H13	OP881092	H6	OP881059	Bimac_3	Sudasinghe et al. (2023)
DZ4859	*Plesiopuntius*	*bimaculatus*	Polonnaruwa: Mahaweli	7.9352 N	81.0112 E	60	OP880898	H20	OP855548	H12	OP881093	H3	OP881048	Bimac_3	Sudasinghe et al. (2023)
DZ4898	*Plesiopuntius*	*bimaculatus*	Rajanganaya: Kala Oya	8.1718 N	80.154 E	63	OP880890	H17	OP855539	H10	OP881082	H6	OP881050	Bimac_3	Sudasinghe et al. (2023)
DZ4899	*Plesiopuntius*	*bimaculatus*	Rajanganaya: Kala Oya	8.1718 N	80.154 E	63	OP880889	H18	OP855540	H11	NA	NA	NA	Bimac_3	Sudasinghe et al. (2023)
DZ5102	*Plesiopuntius*	*bimaculatus*	Thulawelliya, Kanadara Oya: Malwathu Oya	8.5091 N	80.4229 E	66	OP880888	H15	OP855537	H11	OP881081	H6	OP881054	Bimac_3	Sudasinghe et al. (2023)
DZ5103	*Plesiopuntius*	*bimaculatus*	Thulawelliya, Kanadara Oya: Malwathu Oya	8.5091 N	80.4229 E	66	OP880887	H16	OP855538	H10	NA	NA	NA	Bimac_3	Sudasinghe et al. (2023)
DZ3276	*Plesiopuntius*	*bimaculatus*	Peradeniya, Mahaweli basin	7.2574 N	80.5957 E	73	MT732738	H23	MT738922	H15	MT732773	H4	NA	bimac_3	Sudasinghe et al. (2020)
RZM Fish 281	*Plesiopuntius*	*bimaculatus*	Nilwala River, Godapitiya	6.12 N	80.48 E	69	NA	NA	KT736492	H3	NA	NA	NA	bimac_1	GenBank
RZM Fish 375	*Plesiopuntius*	*bimaculatus*	Kelani River, Awissawella	6.96 N	80.19 E	72	NA	NA	KT736493	H16	NA	NA	NA	bimac_3	GenBank
RZM Fish 376	*Plesiopuntius*	*bimaculatus*	Kelani River, Awissawella	6.96 N	80.19 E	72	NA	NA	KT736494	H16	NA	NA	NA	bimac_3	GenBank
PuBB1	*Plesiopuntius*	*bimaculatus*	India: Nelliyampathy, Bharathapuzha	10.552 N	76.717 E	81	done	H32	done	H18	NA	NA	NA	Bimac_3	This study
PuBB2	*Plesiopuntius*	*bimaculatus*	India: Nelliyampathy, Bharathapuzha	10.552 N	76.717 E	81	done	H32	done	H18	NA	NA	NA	Bimac_3	This study
PuBC1	*Plesiopuntius*	*bimaculatus*	India: Nelliyampathy, Chalakudy	10.538 N	76.677 E	82	done	H32	done	H18	NA	NA	NA	Bimac_3	This study
PuBiC1	*Plesiopuntius*	*bimaculatus*	India: Chuchi falls	12.351 N	77.447 E	83	Done	H28	NA	NA	NA	NA	NA	Bimac_3	This study
PuBiC2	*Plesiopuntius*	*bimaculatus*	India: Chuchi falls	12.351 N	77.447 E	83	Done	H28	NA	NA	NA	NA	NA	Bimac_3	This study
PubiE1	*Plesiopuntius*	*bimaculatus*	India: Eastern ghats	18.326 N	82.873 E	84	done	H27	NA	NA	NA	NA	NA	Bimac_3	This study
PuBiKa1	*Plesiopuntius*	*bimaculatus*	India: Kamra	15.372 N	74.413 E	85	Done	H30	NA	NA	NA	NA	NA	Bimac_3	This study
PuBiS2	*Plesiopuntius*	*bimaculatus*	India: Shimoga	13.87 N	75.564 E	86	Done	H29	NA	NA	NA	NA	NA	Bimac_3	This study
PuBiY1	*Plesiopuntius*	*bimaculatus*	India: Yercaurd	11.783 N	78.21 E	87	Done	H31	NA	NA	NA	NA	NA	Bimac_3	This study
PuBiY2	*Plesiopuntius*	*bimaculatus*	India: Yercaurd	11.783 N	78.21 E	87	Done	H31	NA	NA	NA	NA	NA	Bimac_3	This study
